# A non-lipid nucleic acid delivery vector with dendritic cell tropism and stimulation

**DOI:** 10.7150/thno.95267

**Published:** 2024-05-05

**Authors:** Hyung Shik Kim, Elias A. Halabi, Noah Enbergs, Rainer H. Kohler, Fan Fei, Christopher S. Garris, Ralph Weissleder

**Affiliations:** 1 Center for Systems Biology, Massachusetts General Hospital, 185 Cambridge St, CPZN 5206, Boston, MA 02114, USA.; 2 Department of Systems Biology, Harvard Medical School, 200 Longwood Ave, Boston, MA 02115, USA.; 3 Harvard Master's Program in Immunology, Harvard Medical School, 200 Longwood Ave, Boston, MA 02114, USA.

**Keywords:** nucleic acid delivery, nanoparticles, dendritic cells, vaccine, cancer, lymph node

## Abstract

**Rationale**: Nucleic acid constructs are commonly used for vaccination, immune stimulation, and gene therapy, but their use in cancer still remains limited. One of the reasons is that systemic delivery to tumor-associated antigen-presenting cells (dendritic cells and macrophages) is often inefficient, while off-target nucleic acid-sensing immune pathways can stimulate systemic immune responses. Conversely, certain carbohydrate nanoparticles with small molecule payloads have been shown to target these cells efficiently in the tumor microenvironment. Yet, nucleic acid incorporation into such carbohydrate-based nanoparticles has proven challenging.

**Methods**: We developed a novel approach using cross-linked bis succinyl-cyclodextrin (b-s-CD) nanoparticles to efficiently deliver nucleic acids and small-molecule immune enhancer to phagocytic cells in tumor environments and lymph nodes. Our study involved incorporating these components into the nanoparticles and assessing their efficacy in activating antigen-presenting cells.

**Results**: The multi-modality immune stimulators effectively activated antigen-presenting cells and promoted anti-tumor immunity in vivo. This was evidenced by enhanced delivery to phagocytic cells and subsequent immune response activation in tumor environments and lymph nodes.

**Conclusion**: Here, we describe a new approach to incorporating both nucleic acids and small-molecule immune enhancers into cross-linked bis succinyl-cyclodextrin (b-s-CD) nanoparticles for efficient delivery to phagocytic cells in tumor environments and lymph nodes in vivo. These multi-modality immune stimulators can activate antigen-presenting cells and foster anti-tumor immunity. We argue that this strategy can potentially be used to enhance anti-tumor efficacy.

## Introduction

A large number of synthetic nucleic acid analogs have been described as therapeutics, including synthetic nucleotides 1, cyclic dinucleotides (STING activators) [2‑6], oligonucleotides (shRNA, siRNA, miRNA) 7, mRNA 8,9, and DNA 10. The use of these materials has been enabled in part by synthetic DNA technologies 11,12 and a better understanding of nucleic acid modifications on human biology 13,14. These therapeutics generally require delivery vehicles to shield the nucleic acid payload from rapid recognition, degradation, and elicitation of unwanted immune effects via innate viral response mechanisms (TLR, RIG-I, MDA-5, PERK, cGAS/STING pathways). For example, lipid nanoparticles (LNP) have served as efficient delivery systems to deliver modified mRNA as vaccines when injected subcutaneously 15. For systemic delivery and higher or repeat doses, efforts are underway to improve efficacy while limiting toxicity, both from delivery vehicles and payloads 16.

Our group has been interested in developing next-generation myeloid cell therapeutics in cancer 17,18. While we and others have explored numerous approaches [18‑22], it appears that nucleic acids can serve as effective myeloid cells stimulants (via toll-like receptor (TLR), nuclear factor kB (NFkB), stimulator of interferon genes (STING) or other pathways) 23, while larger constructs can potentially be used to impart new functions in myeloid cells 24. To achieve the latter goal, the delivery vehicle will likely need additional functionalities for more efficient cellular delivery, nuclear processing, and synergistic cell stimulation.

We have developed several different carbohydrate-based nano materials with macrophage targeting capabilities [25‑27], some of which are in clinical use. While we have attached nucleotides to the surface of these nanoparticles 28,29, it has not been easy to add additional molecular functionalities that may be required to enhance cellular functionalities of delivery nucleic acids. We hypothesized that strong electrostatic interactions between the particle**'**s surface and the nucleic acids would result in efficient loading. Consecutively, the free cyclodextrin moieties would allow for the adsorption of small-molecule payloads by the strong hydrophobic host-guest inclusion complexation.

Here, we describe the synthesis and characterization of a novel, generic platform for myeloid nucleic acid delivery, using an ionizable amine-containing linker cross-linked with bis-succinyl cyclodextrin (b-s-CD) to achieve i) intracellular nucleic acid delivery, ii) nucleic acid shielding, iii) reasonable circulation times and intra-tumoral accumulation, and iv) adsorption of potent small-molecule immune cell stimulants within the core. The novelty of these constructs arises from their high nucleic acid payload, macrophage affinity, and efficient in vivo stimulation through the synergistic use of small molecule payloads. We show that this modular platform selectively co-delivers immunostimulatory nucleic acid payloads and two immune stimulants (TLR agonists, pathway modulators) to myeloid cells to induce one of the strongest tumoral immune response we have seen with systemically administered adjuvants.

## Results

### Synthesis and characterization

**Figure 1:**
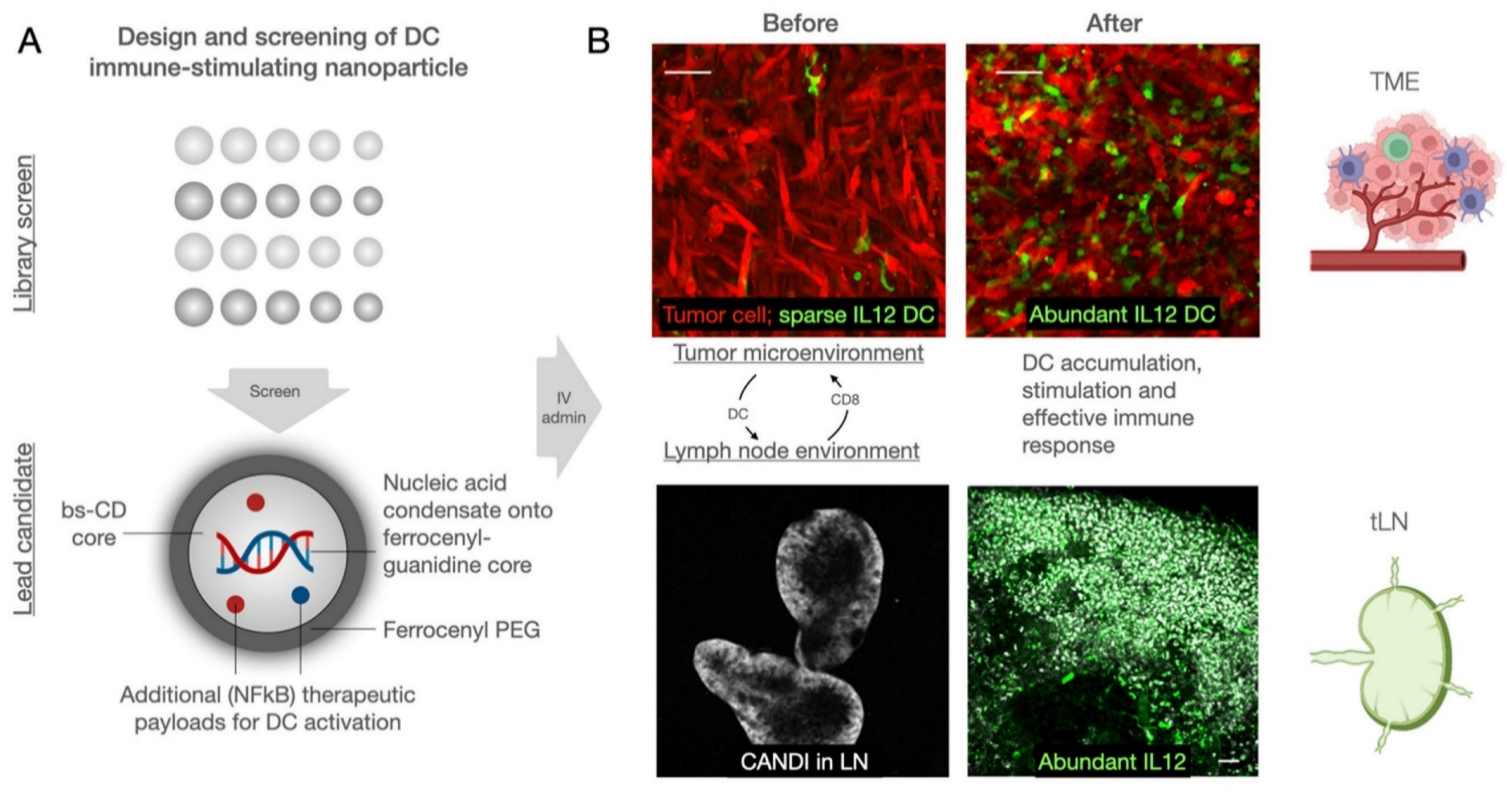
** Design overview of dendritic cell (DC) stimulant. A.** We designed a multifunctional DC targeting and stimulating nanoparticle through an iterative screen. The immunostimulatory nanoconstruct consisted of multiple components, including a cyclodextrin core for immune stimulatory payloads, ferrocenyl-guanidine to charge condensate nucleic acid payloads (poly I:C), and a ferrocene-PEG shell to improve pharmacokinetics. Furthermore, the material contained different NFkB activating payloads to yield DC stimulation, as evidenced by IL-12 induction in vivo. **B.** Following intravenous administration, the nanoparticle effectively accumulated in both the tumor microenvironment and draining lymph nodes, causing remarkable local IL-12 induction (green). Scale bars are 50 µm (top) and 100 µm (bottom).

We designed the nano constructs to allow DC delivery of immunostimulatory nucleic acids and synergistic NFkB pathway modulators. Typical nucleic acid conjugates for such purposes include polyinosinic:polycytidylic acid (poly I:C, a TLR3 agonist) and cyclic dinucleotides (CDN; STING agonist), whereas small-molecule therapeutics include toll like receptor (TLR) 7/8 and cellular inhibitor of apoptosis protein (cIAP) inhibitors. There is emerging evidence that combination treatments 30 result in synergistic immunostimulation with higher immune responses at reduced drug concentration, reducing potential therapy side effects. While lipid nanoparticles have been used to deliver poly I:C and NFkB pathway modulators 31, we reasoned that more stable, polymeric constructs would have practical, pharmacological, and biological advantages. Using bis-succinyl cyclodextrin as a versatile host-guest system, we first optimized linker chemistry and then grafted different components onto the nanoparticles (**Figure 1**).

**Figure 2:**
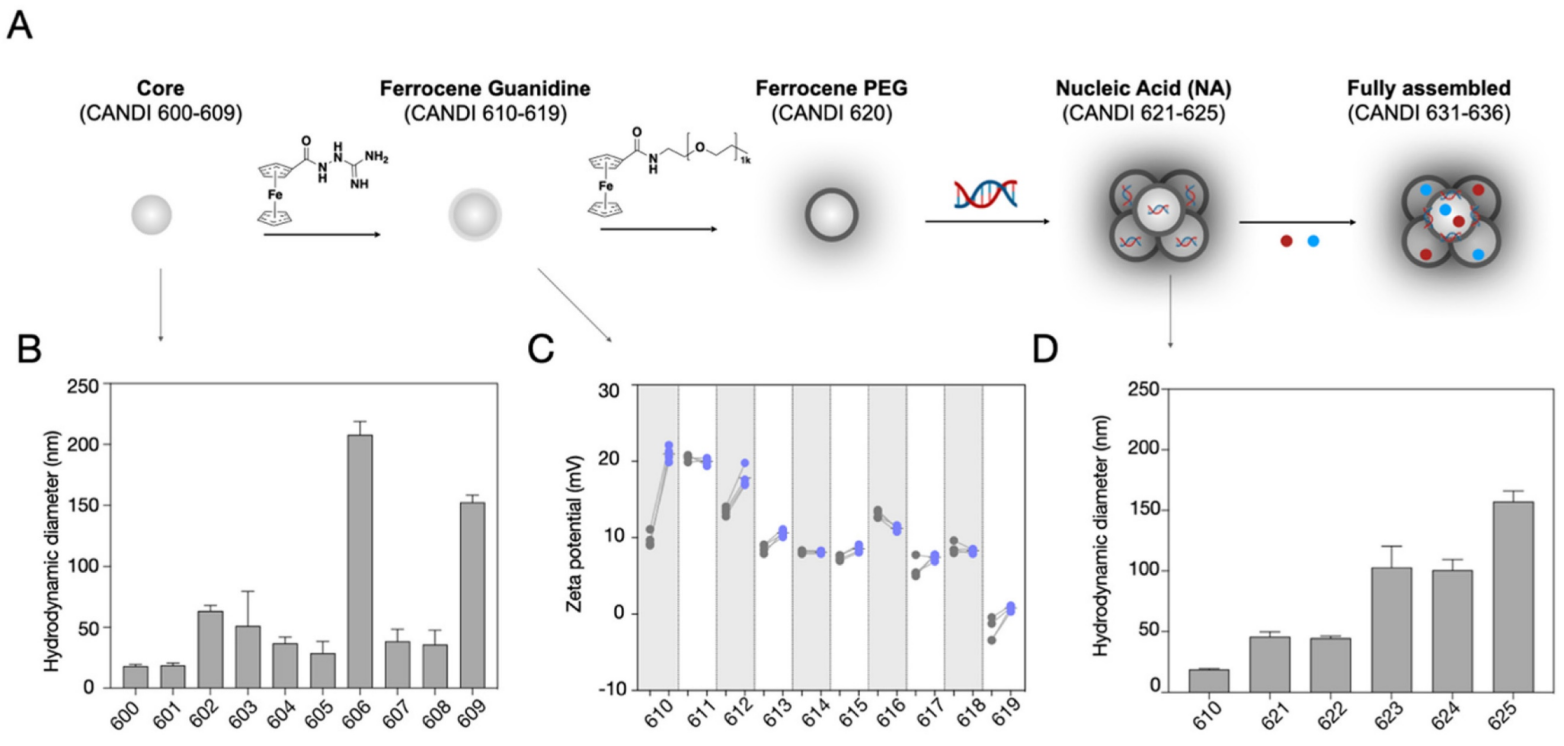
** Synthesis of the nanoparticle platform. A.** Schematic overview of the step-by-step synthesis of the layered the nanoconstructs to allow for i) poly I:C condensation and ii) small molecule immunostimulant loading. The parent compound bis-succinyl-CD was cross-linked with different linkers, summarized in **Figure S1-2**. Through optimization, we identified NAPED as an ideal linker. Subsequently, the nanoparticle core was modified with i) ferrocenyl-guanidine to impart positive charges and ii) ferrocenyl-PEG to improve in vivo circulation times. In a subsequent step, the nanomaterial was loaded with poly I:C and then with the small molecule drug payloads (R848 and LCL-161). **B.** This yielded 10 different core structures (CANDI600-CANDI609) varying in hydrodynamic diameter. **C**. All core materials were subsequently modified with ferrocenyl guanidine to impart a positive charge for nucleic acid condensation. The panel shows the increase in the positive charge for the different nano cores. Note that the NAPED-FG (CANDI610) had a small size and positive charge and thus was chosen for further studies. **D**. Effect of nucleic acid attachment onto CANDI610 by different nucleic acids. Compounds CANDI623 and CANDI625 had hydrodynamic diameters of 100 to 150 nm depending on the amounts and types of poly I:C used (low vs high MW, respectively). Type of nucleic acid attached: CANDI623 = poly IC^Low^; CANDI625 = poly IC^High^; CANDI621 = cGAMP; CANDI622 = mRNA; CANDI624 = plasmid DNA as controls (see **Fig. S6** for optimal loading ratios). Following nucleic acid attachment, additional cyclodextrin moieties were capped with F-PEG. Finally, NFkB modifying small molecules (e.g. R848, LCL161) were used to encapsulate into the core and further yield synergism towards DC stimulation.

**Figure 2** summarizes the different synthetic steps and effects of crosslinker type on CD particle size. Under identical reaction conditions, we compared four different classes of linkers, including polyamines, amino alcohols, amino acids, and charged linkers (**Figure S1**). Dynamic light scattering (DLS) measurements of the purified particles obtained from the cross-linking reactions using linkers *N*1-(2-(4-(2-aminoethyl)piperazin-1-yl)ethyl)ethane-1,2-diamine (NAPED, CANDI600) and betaine (CANDI601) resulted in the smallest cores (<30 nm), whereas using amino alcohols (triethanolamine CANDI603, diethanolamine CANDI604 and ethanolamine CANDI605) and amino acids (L-arginine CANDI602 and L-lysine CANDI608) resulted in medium-sized cores (50-100 nm; **Table S1**). The largest particles were obtained from cross-linking reactions using phosphoserine and taurine (CANDI606 and CANDI609, respectively, >150 nm). The polydispersity index (PDI) for the CANDI600 particle was around 0.2 and slightly higher for the other core analogs (**Figure S1 and S2, Table S1**). We next synthesized a ferrocenyl-guanidine anchoring group for nucleic acids (**Figure S3**), imparting a stable net positive charge on the particle**'**s surface. **Figure 2C** summarizes the zeta potential before and after modification of each of the 10 different materials (CANDI610-619). As expected, there was an increase in positive charge for most of the particles, except for the betaine (611) and p-serine (616).

Having synthesized and characterized the different compounds we next determined which formulations to further explore for subsequent in vitro and in vivo studies. This included size, charge, drug loading (nucleic acid and small molecule immunostimulators) and prior experience with related compounds [27,30,32‑35]. Given the highest transfection efficiency (**Figure S4**), small particle size, low PDI, and effective charge control through FG-guanidine, we selected the CANDI610 nanoparticle platform for subsequent experiments. **Figure 2D** summarizes the effects of nucleic acid condensation onto this CANDI610 particle. The size of the resulting construct increases proportionally with the size of the nucleic acid, as indicated by the respective designations: condensate cGAMP (CANDI621), mRNA (CANDI622), poly I:C^low^ (CANDI623), pDNA (CANDI624), and poly I:C^high^ (CANDI625). Since one of our prime objectives was to deliver the innate immune trigger poly I:C to DC, we selected compound CANDI623 for further experimentation. Two steps remained in the final synthesis loading: loading the nanoconstruct with NFkB modulators and modification of remaining CD units with F-PEG to improve pharmacokinetics (**Figure 2** and **Figure S6**).

**Figure 3:**
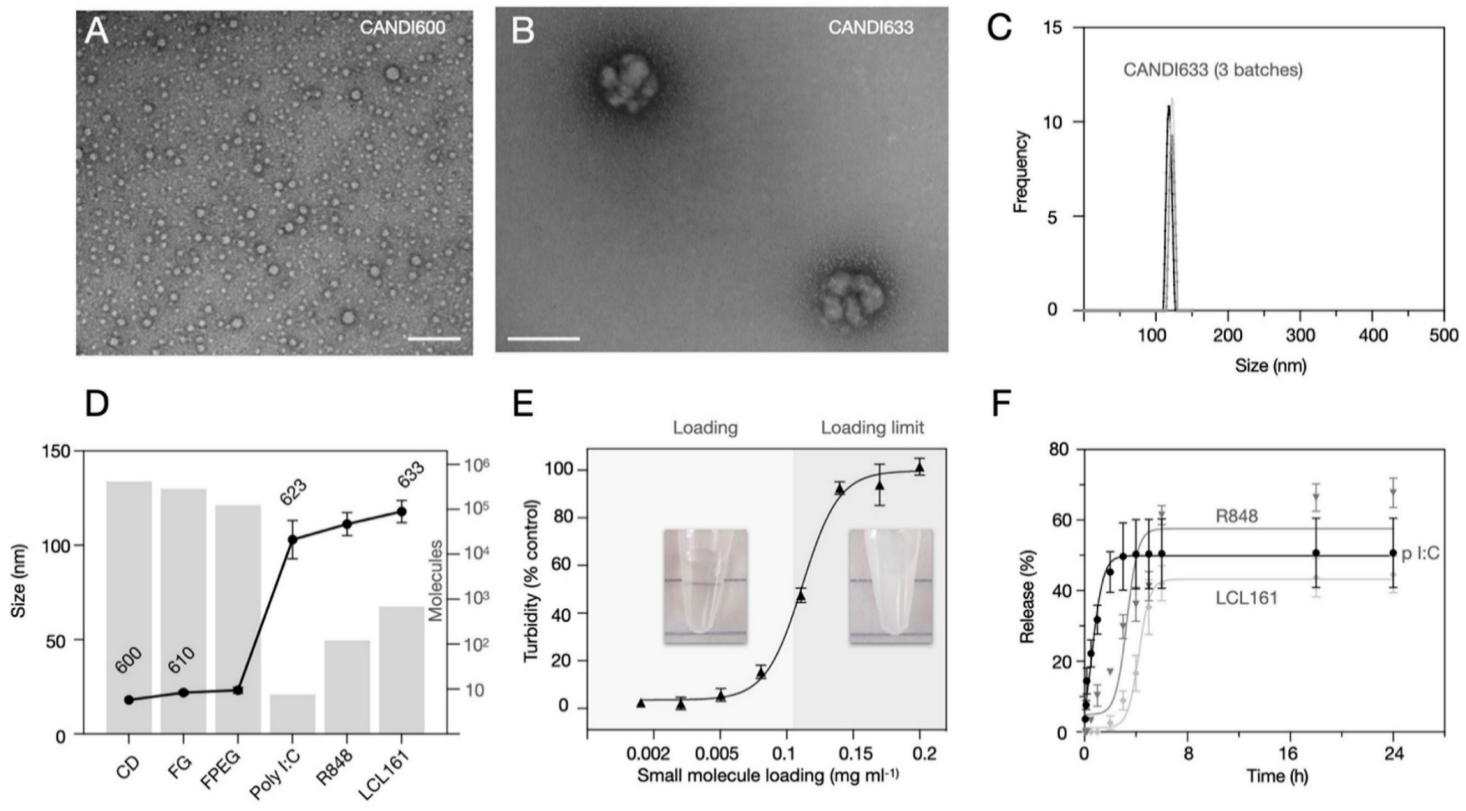
** Composition of lead compound, CANDI633. A**. TEM of the NAPED cross-linked core material (CANDI600) shows single nanoparticles with a mean diameter of ~18 nm (scale bar: 100 nm) **B**. TEM of CANDI633 shows two single nanoparticles. By SEM, the particles have a mean size of ~110-120 nm (scale bar: 100 nm). **C.** Hydrodynamic size of three batches of CANDI633 shows a narrow distribution of particle size and PDI (114 ± 8.7 nm, PDI of 0.229). **D**. Each average CANDI633 consists of an average of 416 molecules of CD, 287,000 molecules of FG, 123,000 molecules of F-PEG, 8 molecules of poly I:C, and 810 molecules of NFkB pathway stimulators (R848, LCL161). **E**. Loading of CANDI622 with small molecule enhancers R848 and LCL161 to yield CANDI633. The particles could internalize up to 0.1 mg of small molecule payload per mg of CANDI. **F.** Release kinetics of poly I:C and small molecule payloads from CANDI633 using a closed-dialysis system with a porous membrane (100kDa and 3 kDa, respectively) in PBS (1×) at 37 °C. The cumulative release (%) of poly I:C was determined by the Ribogreen assay. Each drug was determined as the integrated area of the peak corresponding to each compound (diode array, UV) at multiple points. Shown are results in triplicate for poly I:C (K_off_ = 1.27, t1/2 = 0.84h), R848 (K_off_ = 0.09, t1/2 = 7.8h), and LCL-161 release (K_off_ = 0.07, t1/2 = 9.9h).

**Figure 3** summarizes the characterization studies performed on the lead formulation CANDI633. This included further DLS measurements, transmission electron microscopy (TEM), compositional analysis, loading, and release experiments. CANDI633, the lead compound featuring a core with FG and F-PEG, along with payloads of poly I:C, LCL161, and R848, exhibited a mean size of 120 nm with a well-defined structure (PDI = 0.229) (**Figure 3A**). TEM images revealed that CANDI633 formed globular complexes, particularly with the poly I:C center, resulting in a size of ~110-120 nm. In contrast, CANDI600 remained dispersed as individual nanoparticles with a size of <30 nm, lacking such complexation. In **Figure 3D**, the transformation in size from CANDI600 to CANDI633 is illustrated, emphasizing the composite nature of each unit within the singular CANDI633 structure. We then explored the loading efficiency of small molecules payloads (LCL161 and R848). On average, loading of payloads up to 0.1 mg (0.23 μmol) per mg of CANDI particle yielded stable formulations (**Figure 3E**). We next determined the cumulative drug release for each pharmaceutical component. Poly I:C levels were quantified through RNA detection, and the drugs were analyzed using established liquid chromatography coupled to mass spectrometry (LC-MS) method, employing a closed-dialysis setup. The release pattern was as follows: poly I:C (K_off_ = 1.27, t1/2 = 0.84h), R848 (K_off_ = 0.09, t1/2 = 7.8h) and LCL-161 (K_off_ = 0.07, t1/2 = 9.9h). The release plateaued after 8 h (**Figure 3F**). In additional experiments we also determined the release rates of mRNA (K_off_ = 4.14, t1/2 = 0.18 h) and pDNA (K_off_ = 0.99, t1/2 = 0.71 h), all similar to those of poly I:C, noting their respective dependencies on nucleotide length (**Figure S7**).

### Cellular uptake, stimulation and toxicity

**Figure 4:**
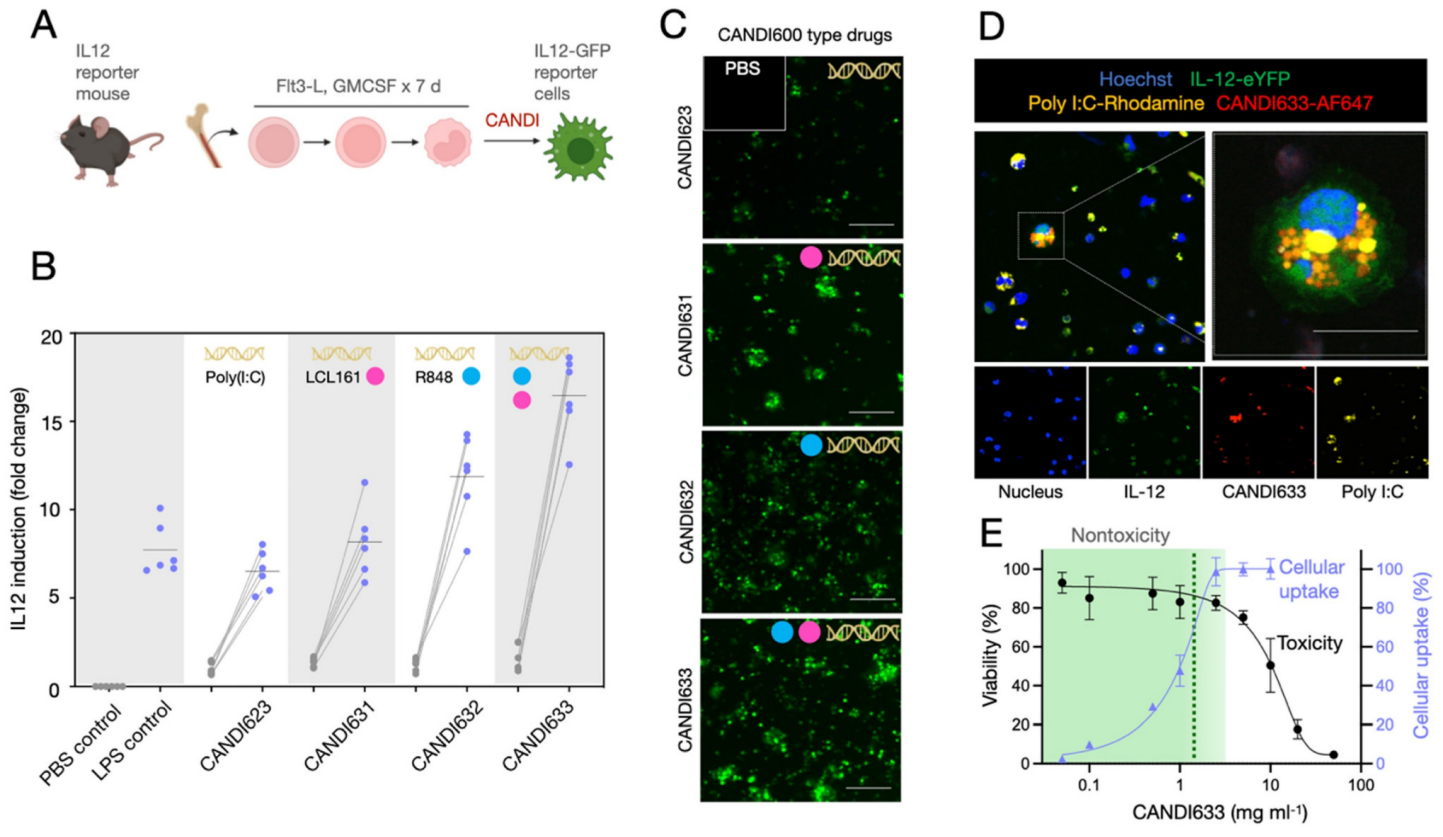
** Cell-based testing of different nanoconstructs. A.** IL-12-GFP reporter mice were used to obtain bone marrow-derived cells. The cells were treated with FLT3L and GM-CSF for seven days to yield DC. In their naive state, the cells had low levels of IL-12-GFP expression. However, upon incubation with the different nano constructs, IL-12 levels increased and were detectable by fluorescent imaging. **B**. Quantitation of screening results. The highest IL-12 induction was observed with CANDI633 yielding nearly 20-fold up regulation of IL-12 in DC. **C**. Representative photomicrographs of IL-12-GFP reporter cells upon stimulation with CANDI-type constructs. **D**. Confocal microscopy of CANDI633 in dendritic cells. The image shows the internalization of the nanoconstruct and IL-12 induction. Scale bar: 20 µm. **E**. Cellular uptake and cytotoxicity of CANDI633. The material was nontoxic to cells, up to 1 mg mL^-1^ (dashed line), well below the in vivo dose (**Figure 5**).

Having synthesized different compounds with poly I:C and NFkB modulators (**Table S1**), we next set out to compare their efficacy in cells (**Figure 4**). These experiments were performed to i) compare cellular uptake, ii) quantitate IL-12 induction in DCs, and iii) evaluate potential cellular toxicity. To enable these comparative studies, we obtained primary bone marrow-derived myeloid cells from IL-12 reporter mice and differentiated them into DCs (**Figure 4A**). Results from these experiments showed that the highest IL-12 induction in DC was achieved with formulation CANDI633, i.e. the nanoparticle that contained dual pathway stimulators in addition to poly I:C (**Figure 4B-C**). Overall, we observed a near 20-fold induction of IL-12, a significantly higher expression when compared to the lipopolysaccharide (LPS) positive control (p < 0.001) 36. The IL-12 levels for the different experimental groups were as follows: 1) No LPS: p < 0.0001; 2) LPS alone (positive control): p < 0.0001; 3) CANDI with poly I:C alone (CANDI-623): p = 0.0006, 4) CANDI with R848 (CANDI-632): p = 0.0005; 5) CANDI with LCL161 (CANDI631): p < 0.0001. Interestingly, we did not observe cellular toxicity at doses < 1 mg mL^-1^. High-resolution confocal microscopy confirmed cellular uptake and IL-12 induction in nearly all cells (**Figure 4D**). High-resolution confocal microscopy confirmed cellular uptake and IL-12 induction in nearly all cells (**Figure 4D**). **Figure S8** summarizes the induction of other chemokine and cytokines as is expected with TLR agonist and other NFkB modulators. Importantly, we did not see a strong cytokine induction with the core nanoparticle (CANDI610), evidence that the iron (Fe) content in the ferrocene is insufficient for stimulation at tested concentrations.

### In vivo properties, IL-12 induction and antitumor efficacy

**Figure 5:**
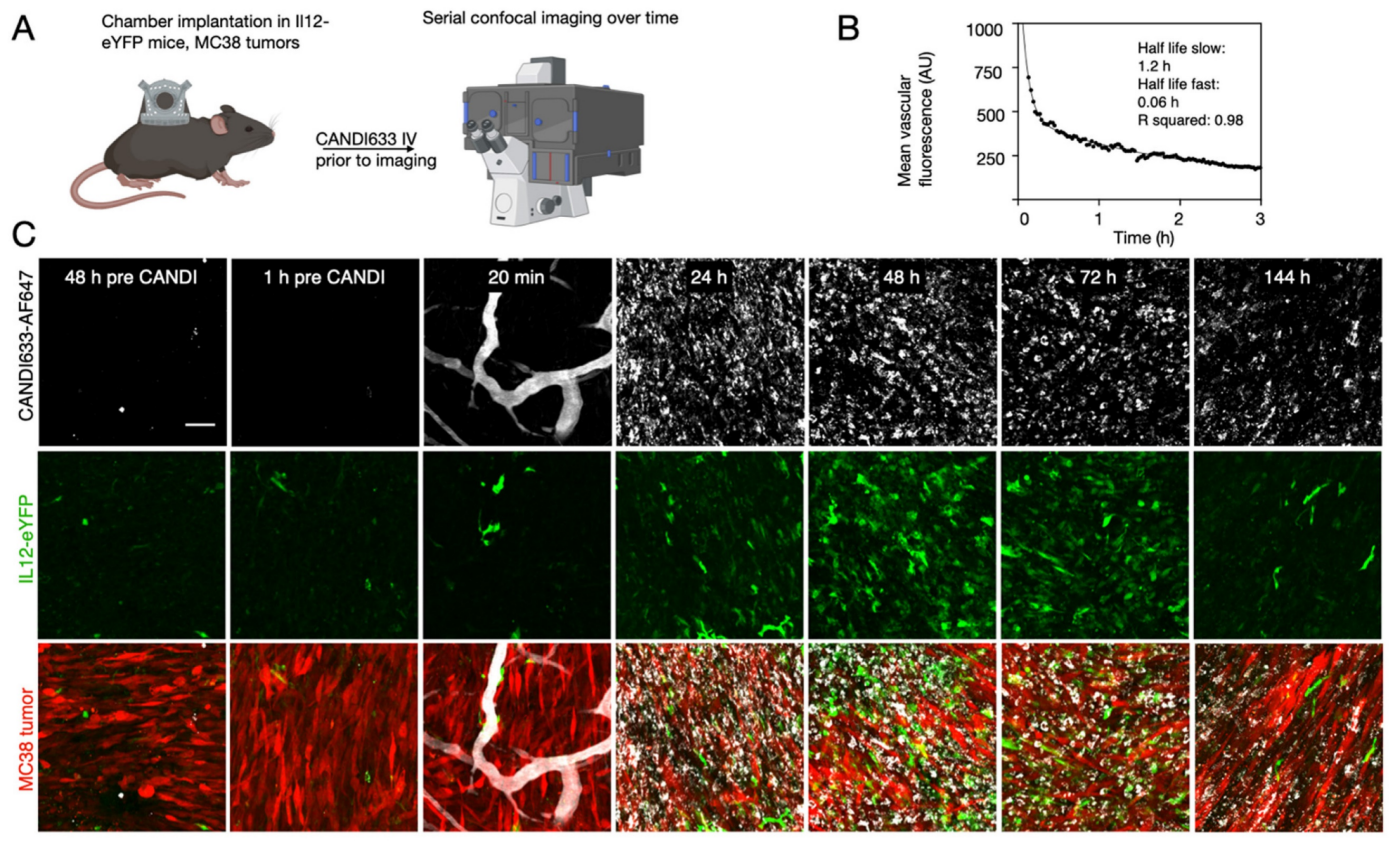
** Intravital microscopy in a live mouse. A.** Experimental set-up. CANDI633, additionally labeled with AF647, was administered systemically while the MC38 tumor microenvironment or lymph nodes were imaged in the IL-12 eYFP reporter mouse. **B**. Blood half-life as determined by serial non-invasive imaging of capillaries in the mouse ear. The slow half-life according to a two-compartment model was approximately 1.2 h (R^2^ = 0.98) **C.** Serial in vivo imaging of the tumor microenvironment before and after IV administration of CANDI633-AF647 (white). Initially, the lead compound is seen in tumor vessels and localizes to mobile dendritic cells within 12-24 h of IV administration. IL-12 induction (green) is seen as early as 24 h after administration and lasts approximately 6 days. MC38-BFP2 tumor cells (red) are shown in the merge channel. For accumulation in lymph node see **Figure S10-S11**. Scale bar: 100 µm.

The lead compound (CANDI633) emerging from the above experiments was next tested in mice using fluorescence imaging and intravital imaging to determine the pharmacokinetics, biodistribution, and cellular uptake (**Figure 5** and **Figure S9-11**). For the intravital experiments, IL-12-GFP reporter or B6 mice were implanted with dorsal window chambers 37, into which MC38-TagBFP2 tumor cells were seeded on day zero. Eight to 10 days later, mice were subjected to intravital imaging during IV administration of a fluorescent version of CANDI633. This setup allowed us to image the initial vascular and cellular distribution of the nano construct in the tumor microenvironment (**Figure 5**). Results from these experiments showed a vascular half-life of approximately 1 h, consistent with the particle size. Several hours after intravenous administration, all CANDI633 was associated with cells in the tumor microenvironment. Dynamic imaging showed that the vast majority and highest amounts of materials accumulated in phagocytic (dextran uptake) antigen-presenting cells (MHCII) and rapidly moving cells, presumably patrolling DCs. Furthermore, cells with high CANDI633 uptake showed high levels of IL-12 induction (**Figure 5**). Similarly, within lymph nodes (**Figure S10**), CANDI633 also accumulated in phagocytic cells and led to high local IL-12 induction.

**Figure 6:**
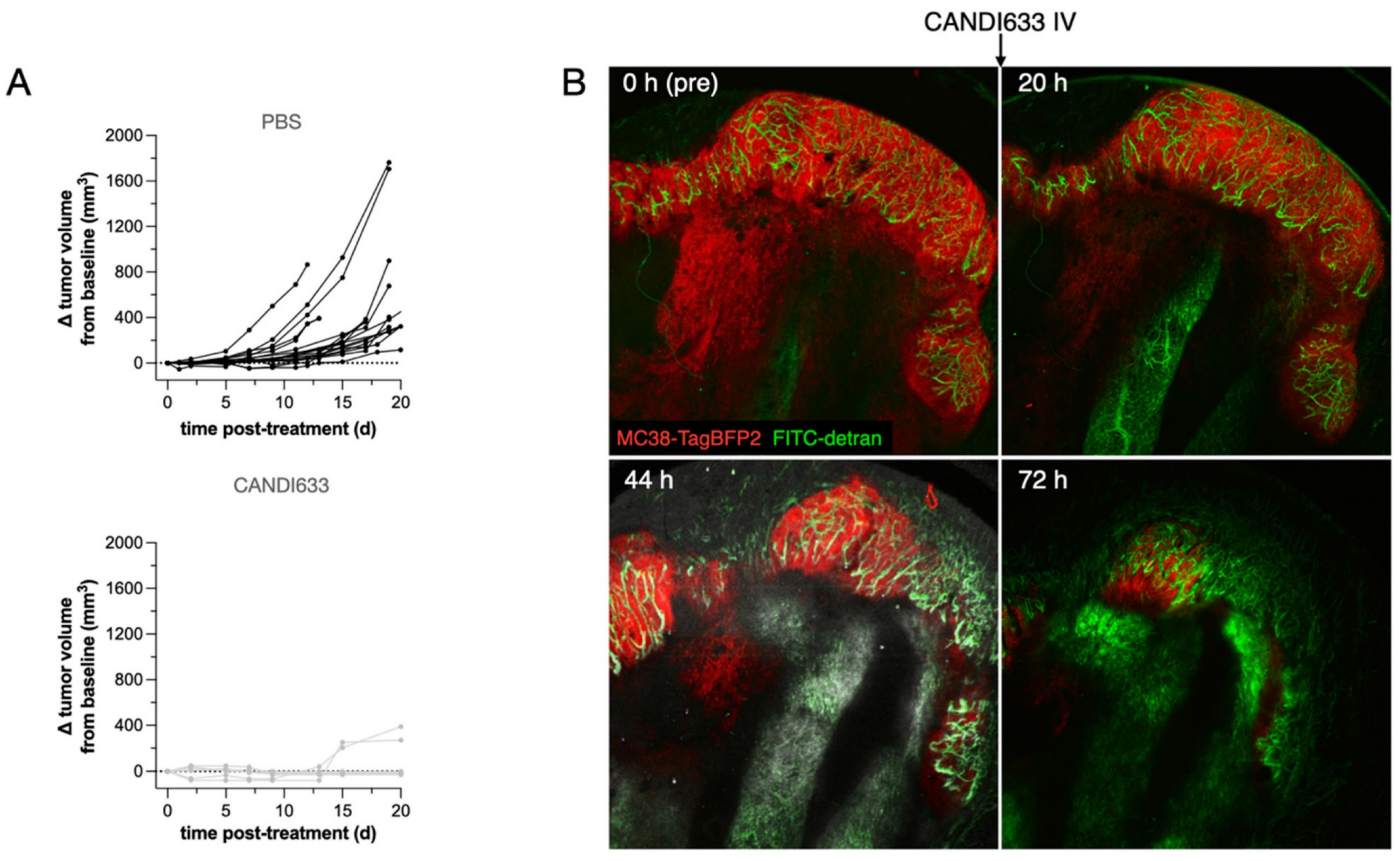
** Anti-tumor efficacy of CANDI633. A.** Tumor growth curves of MC38 tumors in mice injected with control CANDI-633 intravenously (bottom) and control (top). **B**. Serial intravital microscopy experiment of MC38-BFP2 tumor cells (red) before and at multiple time points after IV injection of CANDI-633. FITC dextran was injected to outline the tumor vasculature. Note the massive tumor shrinkage over time following CANDI-633 administration.

To determine the in vivo effects, we also performed anti-tumor efficacy studies using the MC38 model (**Figure 6**). These experiments showed retarded tumor growth and cures with single injections. Furthermore, serial intravital microscopy experiments in the MC38-BFP2 tumor cells (red) showed massive tumor shrinkage over time following CANDI-633 administration which was accompanied by normalization of tumor neovasculature (**Figure 6** and **Figure S12**).

## Discussion

There is an ever-increasing number of nucleic acid therapeutics which emphasizes their potential to treat different human diseases 38. These nucleic acid therapeutics work via different mechanisms, including immune stimulation as shown here, but alternatively could function via gene replacement, addition, inhibition, or editing. Future clinical translation of modified and synthetic RNA/DNA will depend not only on the sequences themselves but also on the delivery construct used to improve nucleic acid stability, targeting, and cellular internalization. Myeloid-derived phagocytic cells are frequently the cell type with which formulations come into contact upon systemic administration. Unfortunately, this can result in acute phase immune responses and concomitant toxicity if not optimized. Conversely, these same myeloid cells often play a fundamental role in disease modulation, and their intentional targeting could be beneficial 18,39. This concept was exploited here in designing next-generation DC immunostimulants based on synergistic targeting of multiple immune stimulatory pathways in these cells.

Here we adapted a bis-substituted cyclodextrin carrier with a natural affinity for phagocytic cells to i) charge couple the innate immune stimulant poly I:C for TLR3 agonism and ii) add small molecules (R848, LCL161) to activate the canonical (TLR7/8 via R848) and non-canonical (cIAP inhibition with LCL161) NFkB pathways 40. We intentionally started the design with bi-succinyl cyclodextrin, which was cross-linked with NAPED to yield a polymeric core structure capable of multiple host-guest interactions. This central core was modified with i) ferrocenyl-guanidine to impart positive charges for poly I:C condensation, ii) small molecule NFkB modulators (R848, LCL161), and iii) ferrocenyl-PEG to extend circulation times. We found this structure to be an efficient delivery system while maintaining DC targeting capabilities in vivo. The CANDI633 construct was stable in PBS at pH 7.4 for, at least 7 days, and could also be lyophilized and resuspended for further pharmacological stability.

**Figure 4** and **5** summarize our in vitro and in vivo observations with CANDI633. As expected, we observed a large induction of IL-12, a key effector cytokine in DCs [40‑43]. Naturally, we also observed induction of other cytokines and chemokine as would be expected with poly I:C and R848/Lcl161 delivery 31. Importantly, we did not observe significant cytokine induction with the ferrocene-guanidine nanoparticle (CANDI610), indication that the Fe concentrations are not sufficient to induce ROS stimulation observed in other ferrocene polymers 44.

While the primary focus of the current research was to develop stable and clinically practical DC immunostimulants, we also show the ability to charge condensate other nucleic acids onto the CANDI600 platform. This naturally would enable the delivery of alternative nucleic acids to cells, including cyclic dinucleotides, plasmids, mRNA, and vectors. Future research will have to investigate transfection efficiencies, stability and in vivo deliveries of future materials. Finally, it is feasible to add peptides and other forms of antigens for vaccination, and this may further expand the utility of this platform.

## Materials and Methods

### Materials

All reagents and solvents were procured from Thermo Fisher, Sigma-Aldrich, BroadPharm or AmBeed and employed without further purification. The small molecules LCL161 (purity: 99.91%), R848 (purity: 99.77%) were obtained from MedChemExpress. The compounds were dissolved in dimethyl sulfoxide (DMSO) as appropriate and were utilized without further processing. MilliQ water was sourced from the Waters filtration system.

### Synthesis

Particle synthesis. We synthesized b-s-CD de novo with a defined degree of substitution (DS) of either 2 or 3 succinyl groups per CD (average DS of 2.5) 33,45. This degree of substitution is narrower than in commercially available products, in which we had observed much broader substitutions ranging from 1-5 succinyls per CD. For the screen of possible linkers, b-s-CD (DS 2.5, 10 mg, 6.99 μmol) was activated with EDC (40.70 mg, 262.2 μmol, 15 eq.) and NHS (15.09 mg, 131.10 μmol, 7.5 eq.) in 240 µl MES-Buffer (50 mM, pH=6.13). The amount of linker used for the synthesis was determined by the number of binding sites for the activated carboxylate. Briefly, N1-(2-(4-(2-Aminoethyl)piperazin-1-yl)ethyl)ethane-1,2-diamine (NAPED) (1.88 mg, 8.74 μmol, 0.5 eq.), betaine (0.5), arginine (1.52 mg, 8.74 μmol, 0.5 eq.), triethanolamine (TEA) (0.86 mg, 5.77 μmol, 0.33 eq.), diethanolamine (DEA) (0.92 mg, 8.74 μmol, 0.5 eq.), ethanolamine (EA) (0.53 mg, 8.74 μmol, 0.5 eq.), phosphoserine (pSer) (1.62 mg, 8.74 μmol, 0.5 eq.), serine (0.92 mg, 8.74 μmol, 0.5 eq.), lysine (1.28 mg, 8.74 μmol, 0.5 eq.), and taurine (1.09 mg, 8.74 μmol, 0.5 eq.) were dissolved in 60 µl MES-Buffer and added drop-wise to the activated b-s-CD, and the reaction was allowed to stir for 24 h. The particles were then purified via filtration (30 k Amicon filter), and then characterized by DLS and Zeta potential before storage at -20 °C. For follow-up experiments, the synthesis for NAPED was scaled up under the same conditions. Nanoparticle characterization by DLS and Zeta potential was repeated for all scaled-up preparations and before in vitro and in vivo testing.

Nuclear magnetic resonance: NMR spectra were recorded on a Bruker Avance UltraShield 400 MHz spectrometer. ^1^H NMR chemical shifts are reported in ppm relative to SiMe_4_ (*δ* = 0) and were referenced internally with respect to residual protons (*δ* = 2.50 for DMSO_d6_ and *δ* = 4.79 for D_2_O). ^13^C NMR chemical shifts are reported in ppm. Coupling constants are reported in Hz. Peak assignments are based on calculated chemical shifts, multiplicity and 2D experiments. IUPAC names of all compounds are provided and were determined using CS ChemBioDrawUltra 15. NMR spectra processing and analysis was performed with MesReNova Research S.L (version 15.0.1).

Liquid chromatography-mass spectrometry (LC-MS): High performance LC-MS analysis was performed on a Waters instrument equipped with a Waters 2424 ELS Detector, Waters 2998 UV- Vis Diode array Detector, Waters 2475 Multi-wavelength Fluorescence Detector, and a Waters 3100 Mass Detector. Separations employed an HPLC-grade water/acetonitrile solvent gradient with one of two columns: XTerra MS C18 Column, 125 Å, 5 μm, 4.6 mm X 50 mm column; Waters XBridge BEH C18 Column, 130 Å, 3.5 μm, 4.6 mm X 50 mm. Routine analysis were conducted with 0.1% formic acid added to both solvents.

Ferrocene-guanidine (FG) synthesis. All synthesis steps were carried out at room temperature. Ferrocenecarboxylic acid (100 mg, 0.43 mmol, 1.0 eq.) and PyBOP (400 mg, 0.77 mol, 1.8 eq.) were dissolved in anhydrous dimethylformamide (DMF, 4 mL). Simultaneously, aminoguanidine hydrochloride (180 mg, 1.6 mmol, 4.4 eq.) was dissolved anhydrous DMF (2 mL) and added dropwise to the aforementioned solution. The reaction proceeded at room temperature for 1 h, followed by the addition of 200 μL of N,N-Diisopropylethylamine (DIPEA). To purify the conjugates, recrystallization was carried out using hexane (20 mL) and diethyl ether (20 mL). Following the reaction, a thorough characterization of the resulting products was conducted through LCMS analysis, revealing specific parameters: retention time of 0.6 min, UV absorbance maxima at 470 nm, and a m/z ratio of 287.23. ^1^HNMR (400 mHz, DMSO_d6_) *δ* = 8.42 (s, 2H), 8.22 (s, 1H), 4.80 (s, 2H), 4.35 (s, 2H), 4.17 (s, 5H), 2.85 (s, 1H). Spectrum in **Figure S14**.

Ferrocene-NHS synthesis. Ferrocenecarboxylic acid (500 mg, 2.17 mmol, 1.0 eq.) and NHS (N-Hydroxysuccinimide, 430 mg (3.74 mmol, 1.7 eq.) were dissolved in anhydrous dichloromethane (DCM, 60 mL). The solution was cooled down with an external ice bath (~0 ºC). Simultaneously, EDC (750 mg, 3.9 mmol, 1.8 eq.) was dissolved in anhydrous DCM (20 mL) and added dropwise to the aforementioned solution. The reaction was left to stir for ~18h at 0°C. Completion of the reaction was monitored by LC-MS. The water-soluble side products were removed by extraction with ammonium chloride (NH_4_Cl) and washed with DCM. The organic phase was dried with magnesium sulfate and the solvents were removed using a rotary evaporator. Following the reaction, thorough characterization of the resulting products was conducted through LCMS analysis, revealing specific parameters: retention time of 1.3 min, UV absorbance maxima at 470 nm, and a m/z ratio of 327.19. ^1^HNMR (400 mHz, DMSO_d6_) *δ* = 7.81 (d, *J* = 8.2 Hz, 2H), 7.35 (d, *J* = 8.0 Hz, 2H), 4.14 (t, *J* = 4.5 Hz, 2H), 3.81 (t, *J* = 4.5 Hz, 2H), 2.44 (s, 3H). Spectrum in **Figure S15**.

Ferrocenyl PEG synthesis. For the synthesis of polyethylene glycol (PEG), Ferrocene-NHS (90 mg, 0.275 mmol, 1.0 eq.) and m-PEG-NH_2_ (MW 1,000, 270 mg, 0.275 mmol, 1.0 eq.) were combined in 6 mL of acetonitrile, followed by the addition of 200 μL of DIPEA. The reaction proceeded for 3 h at room temperature. Purification of the product was achieved using a Biotage SNAP Bio C18 300 Å 4-25 g on a Büchi Pure C-850 FLASHPrep system. Reverse-phase chromatography, unless otherwise specified, was conducted using water (0.1% formic acid) and acetonitrile (0.1% formic acid) as a gradient (20-40 mL per min run). The resulting products were characterized by LCMS analysis, revealing specific parameter: retention time of 1.6 min, UV absorbance maxima at 470 nm, and a m/z ratio of 1222.21. Additional characterization included: ^1^HNMR (400 mHz, D_2_O), ^3^CNMR (101 mHz, D_2_O) as well as 2D-HCOSY NMR spectra shown in **Figure S16**.

### Characterization

Particle size and charge. Particle size and surface charge for all nanoparticle formulations were determined by dynamic light scattering (DLS) and zeta potential measured on a Malvern Zetasizer APS at 5 mg mL^-1^ in PBS (0.5x) and 2 mg mL^-1^ in PBS (0.1x), respectively measured in DTS1170 cuvettes (Malvern) at 25 ºC. All absorbance and fluorescent spectra (e.g. CANDI^AF647^ analogs) were performed with a multimode microplate reader (Tecan, Spark 500) using 96-well transparent bottom black polystyrene microplates (Corning).

Fluorescent CANDI Analogs. Lyophilized CANDI600 was dissolved in carbonate buffer (0.1 M, pH = 8.5), and AF647 succinimidyl ester (ThermoFisher, 2 mg mL^-1^. in DMSO) was added to achieve a final concentration of 50 μM. The reaction was stirred for 45 min at 37 °C in a thermocycler (600 rpm). The labeled nanoparticles were purified by buffer exchange into water against 10 kDa MWCO centrifugal filters (Amicon; 10000 rcf for 5 min; 300 μL water per wash, 3-4×), and the final products were diluted with water or PBS to a final concentration of 50 mg mL^-1^ and filtered through a 0.22 μm sterile filter (VWR) prior to use.

Turbidity assay. CANDI^E^ stock solutions (2.5 mg mL^-1^, 0.5x PBS) were prepared at pH = 7.4, and the small molecules were dissolved in DMSO to prepare payload stocks. Loading of CANDI particles with payloads (0.1-52 mM, respectively, 10% DMSO) was quantified by absorbance scan measurement (λ_abs_ = 400-700 nm) after thoroughly mixing the solutions. The total loading of the payload was determined by the complete loss of absorbance. Data were normalized to payload-free control, and experiments were performed in triplicates (N = 3).

Electron microscopy. CANDI600 and CANDI633 particles were freshly prepared (50 mg mL^-1^, PBS 1x) and diluted with water to a final concentration of 0.1 mg mL^-1^. The particle solution was charged on a TEM grid for 1 min and treated with a 2% aqueous uranyl acetate solution for 15 min, followed by three washing steps with ultra-pure water (x3). Imaging was performed in a transmission electron microscope (JEOL 2100).

NTA assay. The concentration of CANDI633 (35-0.44x10^-6^ g mL^-1^, 0.5x PBS) was determined using a nanoparticle tracking analysis system (Nanosight Malvern). A standard curve with increasing concentrations was constructed for calibration.

Compositional assay. The analysis of ferrocene-derivative composition in CANDI633 was conducted by measuring λ_abs_ = 470 nm using a microplate reader (TECAN). Following the construction of CANDI633, particles underwent five washes with 10 kDa MWCO centrifugal filters (Amicon; 10,000 rpm for 5 min). Concurrently, standard curves for ferrocene-derivatives were established. The collected CANDI633 was subsequently assessed by at λ_abs_ = 470 nm, and the ferrocene-derivative composition was determined using the generated standard curvesThe poly I:C composition in CANDI633 was analyzed using the Ribogreen reagent (Thermo Fisher). Briefly, CANDI633 was diluted in TE (Tris EDTA) buffer, and a poly I:C standard (0-1.25 µg mL^-1^) was concurrently prepared following the manufacturer's guidelines. Subsequently, Ribogreen was added to both the samples. After a 5 min incubation at room temperature, fluorescence intensity was measured (λ_ex_ = 485 nm, λ_em_ = 515 nm). To confirm the stable interaction of poly I:C with CANDI633, 100 kDa MWCO centrifugal filters (Amicon; 10,000 rpm, 5 min) were utilized. Poly I:C concentration was quantified using standard curves, and molar concentration was determined through dsRNA mass conversion methods.

The composition of R848 and LCL161 in CANDI633 was analyzed using LCMS. To confirm drug loading on CANDI633, a preliminary test was conducted with three study groups: R848 and LCL161 in DMSO, CANDI633 in DMSO, and CANDI633 in water. Non-interlocked drugs were removed using 3 kDa MWCO centrifugal filters (Amicon; 10,000 rpm, 20 min), and examined by LCMS. The CANDI633 construct in water demonstrated drug interlocking, highlighting the host-guest interaction with b-s-CD. Upon confirming drug interlocking on CANDI633, standard curves for R848 (0-400 nM) and LCL161 (0-500 nM) were established by measuring the area under the curve (AUC). The composition of each drug was determined by applying AUC to the standard curves and calculating the results. Unit quantification involved multiplying Avogadro's number and dividing by the number of particles, as measured by NTA analysis. All experiments were performed in triplicates (N = 3).

Drug Release Kinetics. Kinetics of drug release was performed in a closed dialysis setup employing a 3 kDa molecular weight cutoff membrane (Pur-A-Lyzer Midi Dialysis Kit). Solutions of CANDI600 (200 mg) were loaded with LCL-161 (15 µM) and R848 (2.8 µM) in PBS (1×, 1 mL) containing 10% DMSO and were dialyzed against PBS (1×, 6 mL) at 37 °C under constant stirring (600 rpm). The percentage of eluted molecules was quantified by analysis of the liquid chromatographs at different time points (t = 0, 0.05, 0.1, 0.2, 0.5, 1, 2, 3, 4, 5, 6, 18, 24, 48, and 72 h), injecting a total of 100 μL aliquots into an LC-MS and subsequently replacing the system with an additional 100 μL of PBS. Each payload was identified by its distinctive retention time (R848 = 0.78 min and LCL- 161 = 1.01 min) and mass-to-charge ratio (ES-: R848 = 313 and LCL-161 = 499). The cumulative drug release was determined as the ratio of the integrated area under the curve for each eluted peak to the total area under the curve of chromatographs obtained from the non-dialyzed solutions. All experiments were performed in triplicates (N = 3).

Poly I:C Release Kinetics. Kinetics of Poly I:C release was performed in a closed dialysis setup employing a 100 kDa molecular weight cutoff membrane (Pur-A-Lyzer Midi Dialysis Kit). Solutions of CANDI600 (10 mg) were loaded with Poly I:C (62.5 µg), LCL-161 (15 µM) and R848 (2.8 µM) in PBS (1×, 1 mL) containing 10% DMSO and were dialyzed against PBS (1×, 6 mL) at 37 °C under constant stirring (600 rpm). The percentage of eluted molecules was quantified by analysis of the liquid chromatographs at different time points (t = 0, 0.05, 0.1, 0.2, 0.5, 1, 2, 3, 4, 5, 6, 18, 24, 48, and 72 h), injecting a total of 100 μL aliquots into prepared RNA detection kit (Ribogreen) following the manufacturer's guidelines. After a 5 min incubation at room temperature, fluorescence intensity was measured (λ_ex_ = 485 nm, λ_em_ = 515 nm). Poly I:C concentration was quantified using standard curves. All experiments were performed in triplicates (N = 3).

### Cell models

Bone marrow-derived cells. Murine bone marrow-derived cells were isolated from IL-12-eYFP reporter mice for flow cytometry and live-cell microscopy analyses. To obtain the whole bone marrow, femurs and were prepared and flushed with sterile PBS using syringes and a 28-gauge needle. RBC Lysis Buffer (BioLegend) was then used according to the manufacturer**'**s instructions to lyse red blood cells. The remaining cells were counted using a Neubauer chamber and seeded into either transparent (NEST, flow cytometry analysis) or black (Ibidi, glass bottom for imaging) 96 well plates at a density of 1.25 x 10^5^ cells per well. Bone marrow-derived macrophages (BMDMs) were differentiated by adding 50 ng mL^-1^ recombinant murine M-CSF (BioLegend) to cell culture media for 7 days. Bone marrow-derived dendritic cells (BMDCs) were differentiated by adding 20 ng mL-1 recombinant murine GM-CSF (BioLegend) and 200 ng mL-1 FLT-3L to cell culture media for 7 days. New media was added every 3-4 days.

Immortalized cell lines. The immortalized murine bone marrow-derived macrophages (iMACs) 46 were acquired from Charles L. Evavold (Ragon Institute, Harvard University) and used to assess toxicity. Briefly, iMAC cells were plated and grown in Dulbecco**'**s Modified Eagle Medium (DMEM, Corning) supplemented with 10% Fetal Bovine Serum (Corning) and 1% Penicillin Streptomycin (Corning) at 37 °C and 5% CO_2_ and MC38 cells were cultured in Iscove**'**s Modification of DMEM (Corning). Upon reaching confluency, cells were split using 0.05% Trypsin / 0.53 mM EDTA (Corning), and all* in vitro* assays were performed after the cells reached 90% confluency. Prior to cell culture application, all CANDI preparations were filtered through a 0.22 μm sterile filter (VWR).

### Microscopy

Live-cell microscopy was performed to determine the cytokine production in bone marrow-derived macrophages of reporter mice. Harvested cells were treated with various combinations of test compounds. Cells were imaged in a 96-well plate. Before imaging, cells were stained with Hoechst 33342 (15 μg mL^-1^, Thermo Fisher) according to the manufacturer**'**s protocol. Fluorescence microscopy was performed using an IX81 inverted fluorescence microscope (Olympus, Tokyo, Japan) equipped with a motorized stage (Renishaw, Wotton-under-Edge, England, UK) and fitted with an ORCA-Fusion Digital CMOS camera (Hamamatsu Photonics, Hamamatsu, Japan). Using CellSens Dimension 3.1.1 software (Olympus), multiple fields of view were acquired for each sample with a UPlanSApo ×10 (numerical aperture (NA) 0.75, Olympus) or a UPlanSApo ×40 air objective (NA 0.95, Olympus). In addition to brightfield, four fluorescent channels were acquired DAPI (345/455 nm), GFP (489/508 nm), YFP (550/565 nm), CY3 (550/565 nm), and CY5 (625/670 nm) were excited with the appropriate optical filters.

### Toxicity

Toxicity. iMACs were seeded in 96 well plates at a density of 15 x 10^3^ cells per well and incubated for 24 h at 37 °C and 5% CO_2_ before use. Stock solutions of different CANDI nanoparticles (**Table S1**) were prepared and then diluted in cell culture medium to desired concentrations (0.09 μg mL^-1^ to 10 mg mL^-1^, DMSO 0.5%). Cells were incubated for 2.5 h with nanoparticles before the medium was exchanged. Cells were further incubated for 48 h at 37°C and 5% CO_2_. Subsequently, cells were thoroughly washed with PBS buffer. A solution of 10% Prestoblue^TM^ (Invitrogen) in serum-containing media (220 µL) was added to each well and incubated at 37°C for 2 h. Cell viability was determined by measuring fluorescence intensity at 570 nm using a fluorescence microplate reader (TECAN) after the 48 h incubation period.

Comprehensive cytokine array panel. The mouse cytokine array kit (R&D Systems, ARY006) was utilized for the parallel determination of the selected mouse cytokines levels produced by BMDMs. Following treatment with either CANDI610 or CANDI633, the cell culture supernatant was centrifugated to remove any suspended cells. Subsequently, 1 ml of the clarified supernatant was utilized in accordance with the manufacturer**'**s protocol.

### Mouse models

Experiments were approved by the MGH Institutional Animal Care and Use Committee (IACUC) and were performed in accordance with MGH IACUC regulations (2013N000157). IL-12p40-eYFP mice (N = 6) were used for primary myeloid cell harvesting and IL-12 induction experiments as well as intravital imaging (N=4). Female C57BL/6J mice (N = 4) were utilized for MC38 tumor imaging studies by intravital microscopy. All mice were either bred IL-12 or obtained from Jackson Laboratory (Stock# 000664) and housed under specific pathogen-free conditions at the Massachusetts General Hospital.

### Intravital imaging

Mice bearing dorsal window chambers with MC38-mTAG-BFP2 cells were imaged to determine the kinetics of IL-12 induction in the tumor microenvironment. Dorsal window chambers were implanted into mice using well-established techniques 37. Fluorescent tumor cells (MC38-mTAGBFP2) were implanted in the window chambers as previously described 47,48 and allowed to grow for 8-10 days before imaging experiments, with tumor growth monitored regularly.

All confocal images were collected using a customized Olympus FV1000 confocal microscope (Olympus America). A 2x (XLFluor, NA 0.14), a 4x (UPlanSApo, NA 0.16), and an XLUMPlanFL N 20x (NA 1.0) water immersion objective were used for imaging (Olympus America). Tumor cells (MC38-TagBFP2), fusion-protein IL-12-GFP, and CANDI^AF647^ were excited sequentially using a 405 nm, a 473 nm, and a 633 nm diode laser in combination with a DM-405/488/559/635 nm dichroic beam splitter. Emitted light was further separated by beam splitters (SDM-473, SDM-560, and SDM-640) and emission filters BA430-455, BA490-540, BA575-620, and BA655-755 (Olympus America). Confocal laser power settings were carefully optimized to avoid photobleaching, phototoxicity, or tissue damage. Fiji (ImageJ, 2.3.0/1.54d) was used for image analysis.

### Statistics

All statistical data analyses were performed using GraphPad Prism 9 software, and results are expressed as mean ± standard deviation. For normally-distributed datasets, we used 2-tailed Student's t test and one-way ANOVA followed by Bonferroni**'**s multiple comparison test. When variables were not normally distributed, we performed non-parametric Mann-Whitney or Kuskal-Wallis tests. p values > 0.05 were considered not significant (n.s.), p values < 0.05 were considered significant.

## Supplementary Material

Supplementary figures and table.
