# Supplementary material for: A non-lipid nucleic acid delivery vector with dendritic cell tropism and stimulation
Source: Theranostics. 2024 May 5;14(7):2934–45. doi: 10.7150/thno.95267 (PMC11103498; doi:10.7150/thno.95267)
Supplement: Supplementary file 1 — Supplementary figures and table. [file thnov14p2934s1.pdf]

Supporting information

Hyung Shik Kim*, Elias A. Halabi*, Noah Enbergs, Rainer H. Kohler, Fan Fei, Christopher S. Garris, Ralph Weissleder#

[1] Center for Systems Biology, Massachusetts General Hospital, 185 Cambridge St, CPZN 5206, Boston, MA 02114,

[2] Department of Systems Biology, Harvard Medical School, 200 Longwood Ave, Boston, MA 02115

[3] Harvard Master's Program in Immunology, Harvard Medical School, 200 Longwood Ave, Boston, MA 02114

* equal contribution

#R. Weissleder, MD, PhD
Center for Systems Biology
Massachusetts General Hospital
185 Cambridge St, CPZN 5206
Boston, MA, 02114
617-726-8226
rweissleder@mgh.harvard.edu

Keywords: DNA delivery, nanoparticles, myeloid cells, macrophages, vaccine

Figure S1: Composition of CANDI cores. A. The core structure was constructed with bis-succinyl cyclodextrin moieties cross linked with a number of different linkers (see **Figure S2** for additional detail). A total of 10 different core structures were synthesized and analyzed (**Figure S3**). From this screen, NAPED emerged as the preferred linker for subsequent particle assembly and nucleic acid condensation.

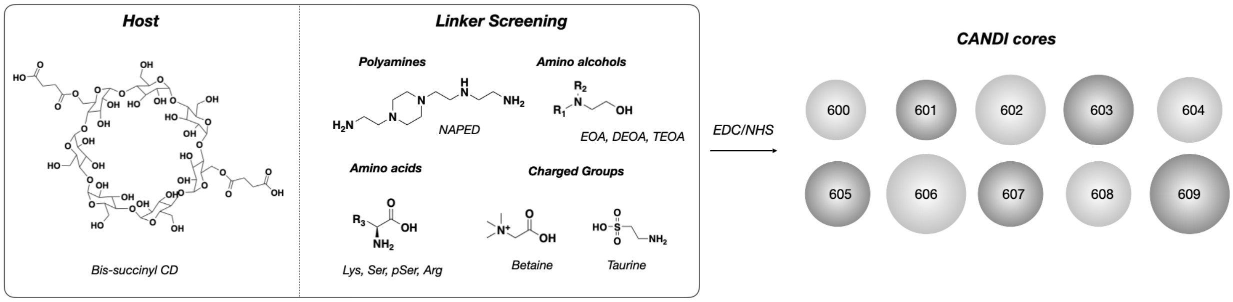

Figure S2: Overview of linkers tested. b-s-CD was cross-linked with 10 different linkers shown, and particle sizes and zeta potential were determined (**Figure 2 and Figure S4**).

Linkers	Abbreviations	Structure	CANDI #
N^1-(2-(4-(2-Aminoethyl)Piperazin-1-yl)ethyl)Ethane-1,2-Diamine	NAPED	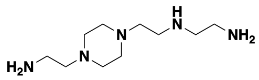	600
Betaine	Bet	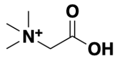	601
Arginine	R	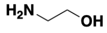	602
Triethanolamine	TEA	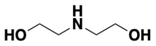	603
Diethanolamine	DEA	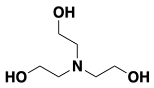	604
Ethanolamine	EA	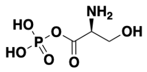	605
Phosphoserine	pS	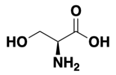	606
Serine	S	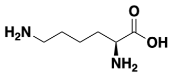	607
Lysine	K	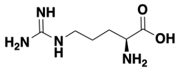	608
Taurine	Tau	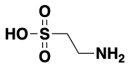	609

Figure S3: Synthesis of ferrocenyl-guanidine. A. Synthetic scheme. Ferrocenyl-guanidine (FG) was synthesized by coupling carboxyl ferrocene with aminoguanidine. **B.** Picture of the recrystallized reaction product obtained at 30% yield. **C.** Chromatographic analysis. LCMS-ELSD chromatograms, highlighting the comparison between ferrocene carboxylic acid and ferrocene-guanidine. **D**. Mass spectrometry insight. ES Ionization (Positive Ion Mode) reveals the relative intensity of ferrocene-guanidine (m/z 287.05). **E.** Spectral confirmation. UV-Vis spectrum showcasing specific peaks at 326 nm and 470 nm, providing clear evidence of the successful conjugation between ferrocene and aminoguanidine. **F.** Surface charge transition of CANDI611 after loading with ferrocene-guanidine.

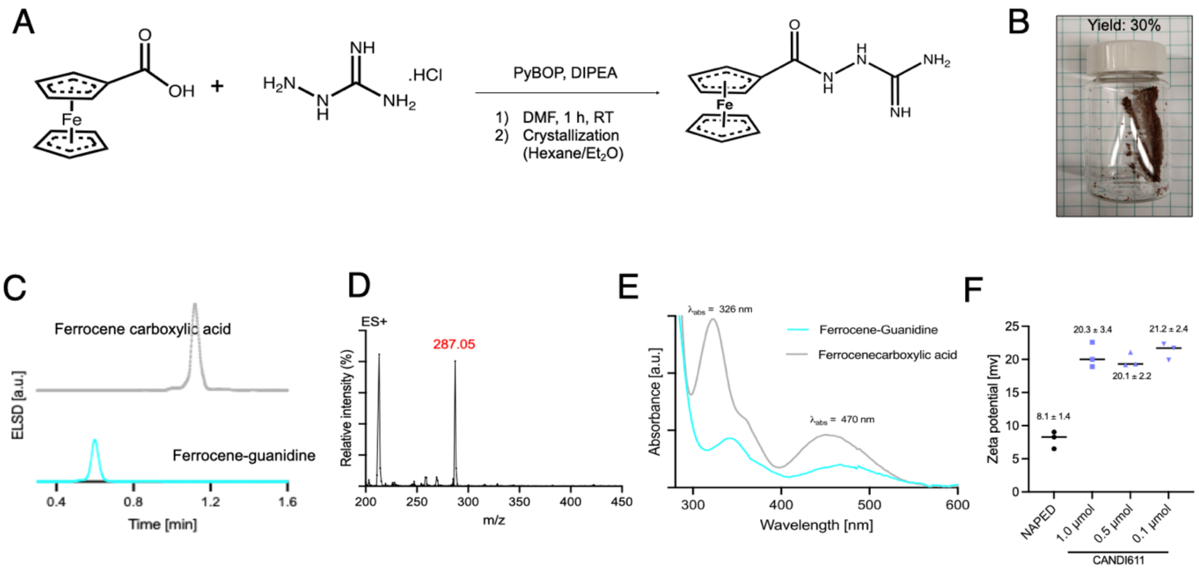

Figure S4: Transfection efficiency of different CANDI nanoparticles. A. Transfection efficiency screen of mRNA-GFP loaded CANDI constructs. Note the highest transaction efficiency of the NAPED cross-linked CANDI610, the reason for which it was chosen as a platform for subsequent experiments. **B.** Quantitation of GFP transfection efficiency of BMDM. **C.** Effect of nucleic acid loading onto CANDI610. The graph describes the zeta potential of NAPED-FG nanoconstructs loaded with different types and amounts of nucleic acids (see **Figure 2D** and **S5** for the hydrodynamic diameter of fully loaded nano constructs). cGAMP = CANDI621; mRNA = CANDI622; Poly IC^{Low} = CANDI623; plasmid DNA = 624; Poly IC^{High} = CANDI625. Note good loading at nanoparticle/nucleic acid ratios of 4 and above.

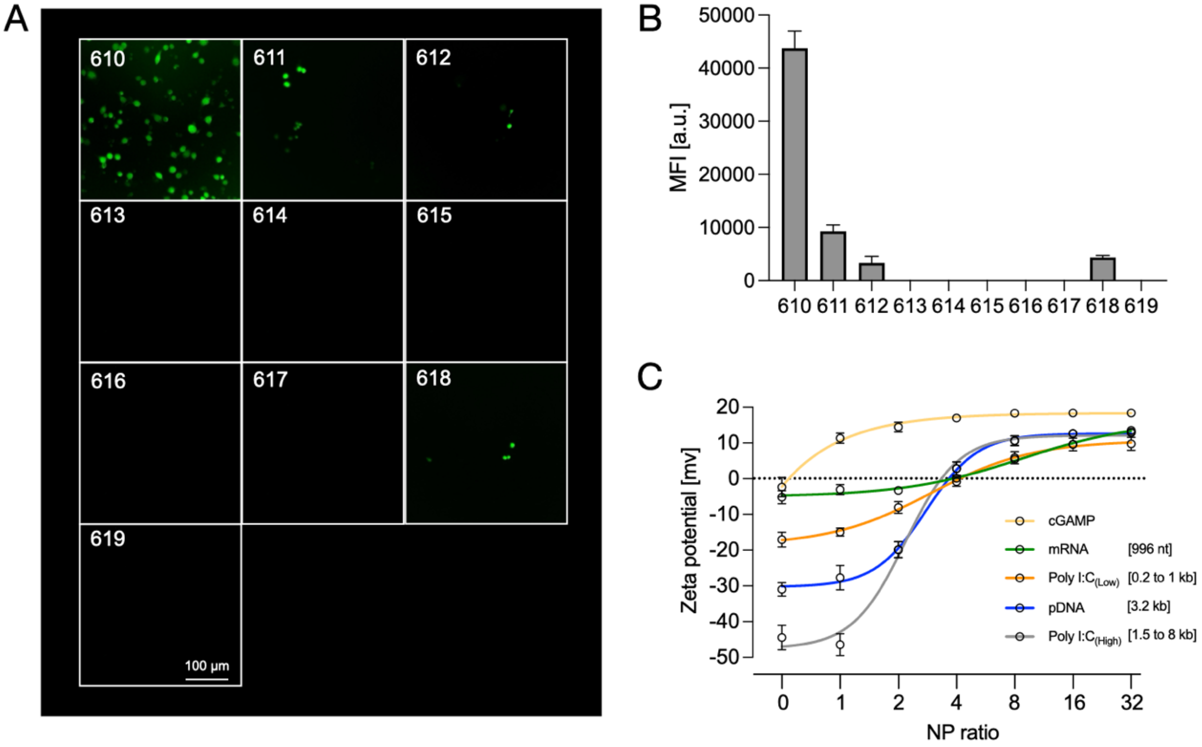

Figure S5: Size and polydispersity index of different CANDI cores. A. PDI and size of different nanoparticles synthesized. For particle design, we accepted a PDI < 0.4 and mean sizes < 50 nm. **B.** PDI for different nucleic acid conjugates.

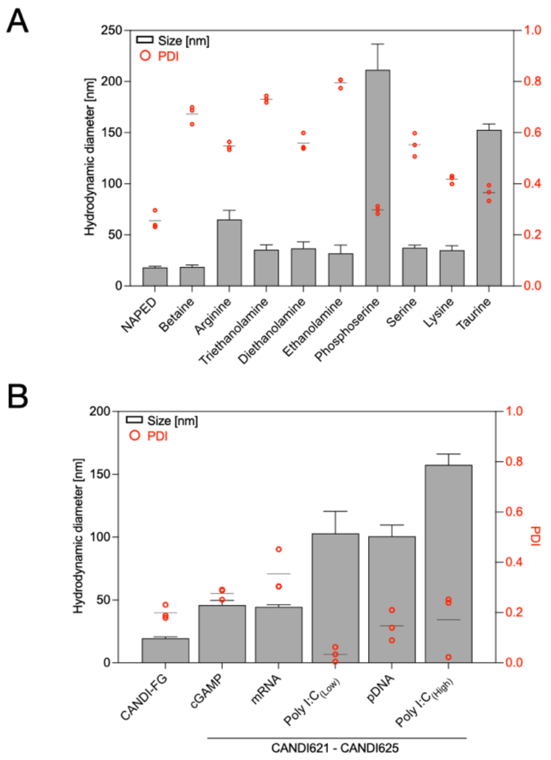

Figure S6: Synthesis of F-PEG. A. Synthetic scheme. Ferrocenyl-PEG was synthesized by coupling ferrocene NHS ester with mPEG-NH2. **B.** Chromatographic analysis. LCMS-ELSD chromatograms, highlighting the comparison between ferrocene-NHS ester and ferrocene-PEG. **C**. Mass spectrometry insight. ES **I**onization (Positive **I**on Mode) reveals the relative intensity of ferrocene-PEG (m/z 1213.31). **D.** Spectral confirmation. UV-Vis Spectrum show peaks at 326 nm and 470 nm, proving the conjugation between ferrocene and PEG.

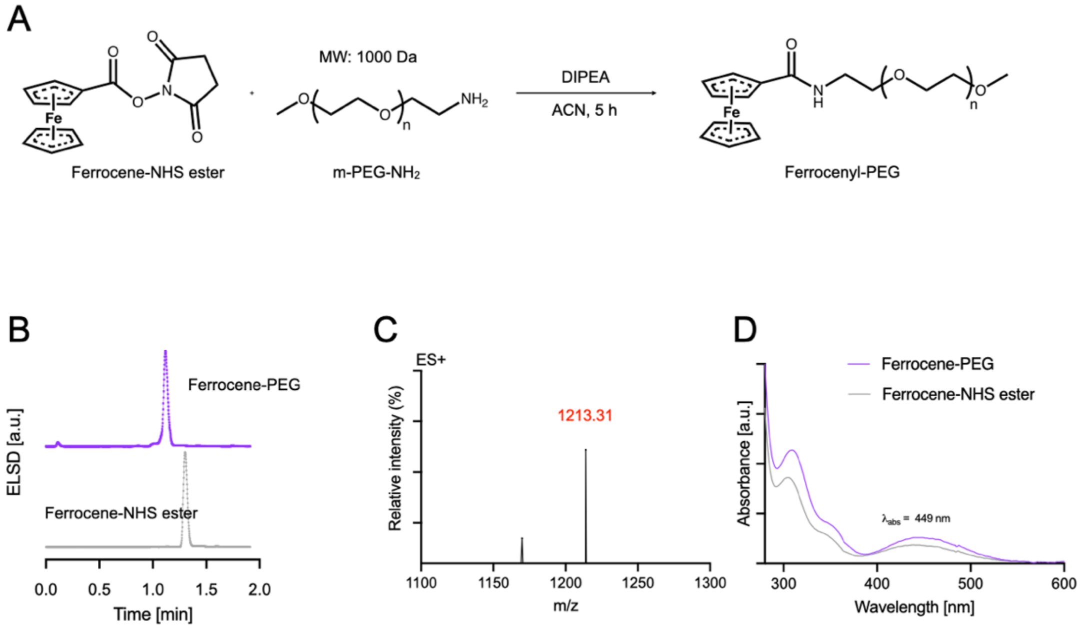

Figure S7: Releasing profile of nucleic acid from CANDI. Cumulative release profile of nucleic acids from CANDI, illustrating the release kinetics of **A.** mRNA and **B.** pDNA. Each experiment was conducted in triplicate and analyzed independently.

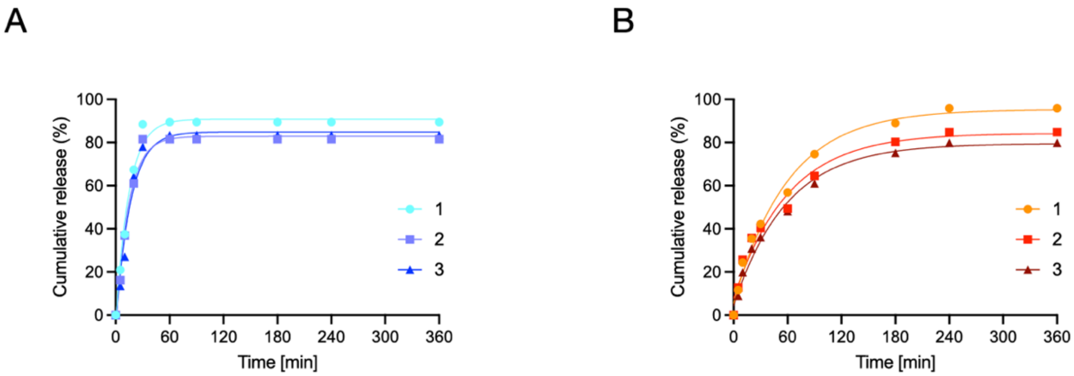

Figure S8: In vitro drug effects. In order to determine whether cytokine production in bone marrow-derived DC was specific to the nanoparticle delivery system, we compared empty (CANDI610) and fully assembled nanoparticles (CANDI633). Note the remarkable cytokine production with CANDI633 in a typical ISG (interferon-stimulated gene) pattern.

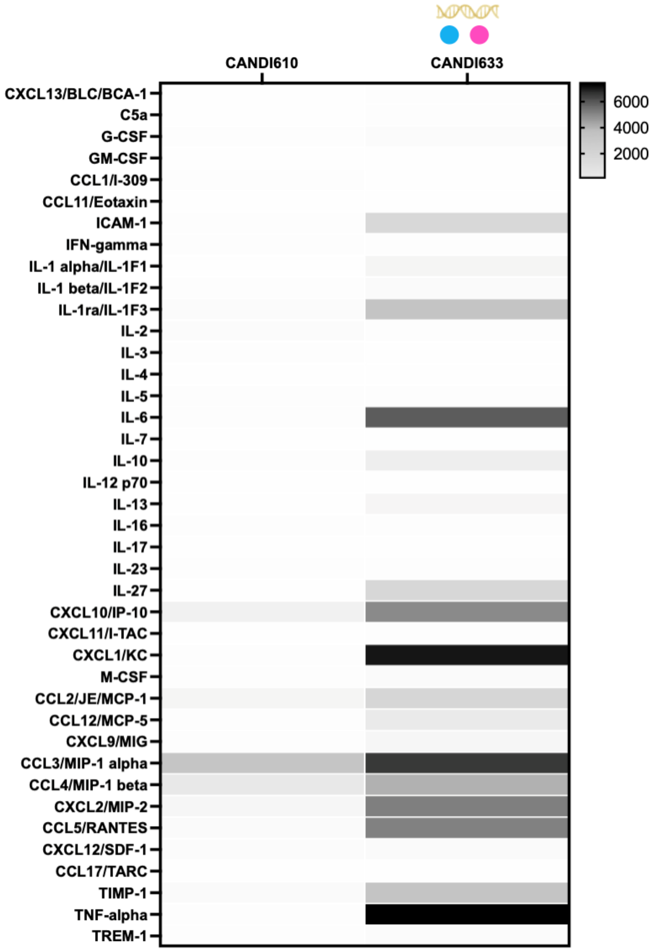

Figure S9: In vivo distribution. To determine the in vivo distribution of CANDI-633, AF647 labeled material was injected into mice and tissues were removed 24 h later for fluorescence measurements. A. Representative images of excised organs and their fluorescence intensity. **B.** Distribution of IV injected CANDI-633-AF647 shows high accumulation in tumors. C. CANDI-647-AF647 also accumulated in lymph nodes, both tumor draining (TdLN) and non-tumor draining (NdLN). For microscopic distribution in lymph nodes, see **Figure S11**.

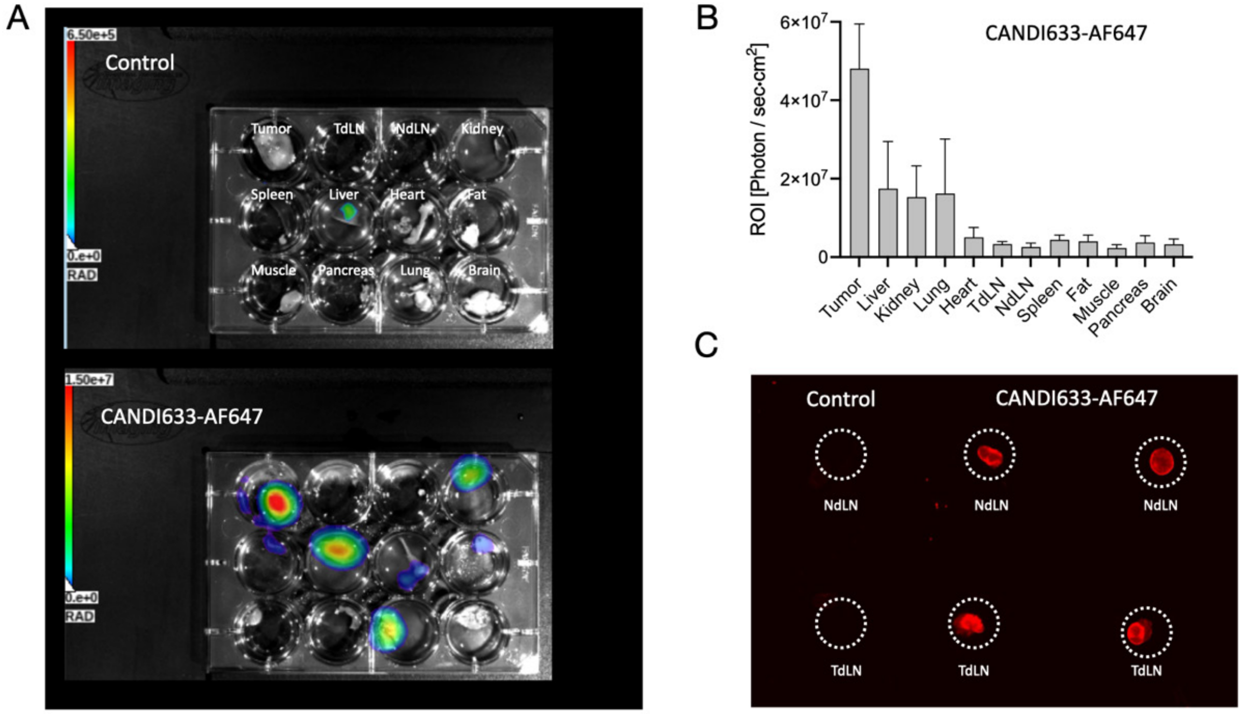

Figure S10: Lymph node accumulation. Intravital images (**A.** 20 x objective; **B.** 40 x objective) of tumor-draining lymph node 24 h following the IV administration of CANDI633-AF647 (white). Note the extensive accumulation of the material throughout the lymph nodes. The green channel shows the IL-12eYFP signal, which is largely co-localized with the CANDI-647 signal.

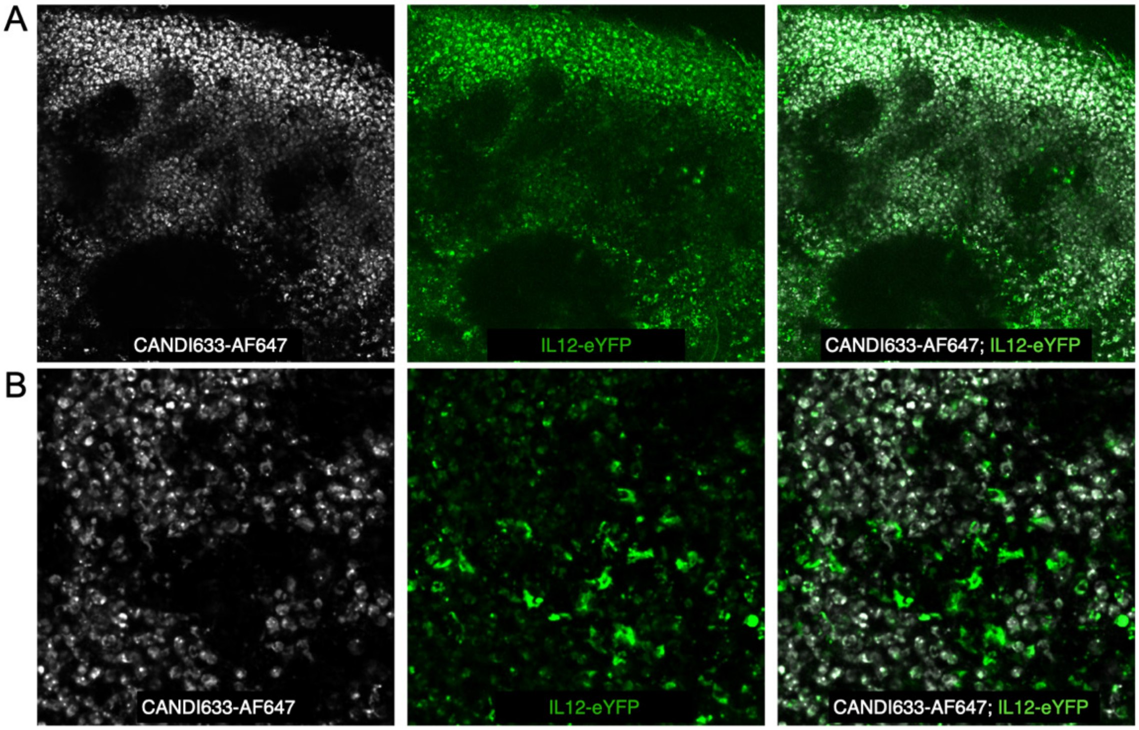

Figure S11: Accumulation of CANDI-633 in dendritic cells. A. Flow cytometry of lymph node of a mouse injected with CANDI-633-AF647. This panel shows that 65.5% of all Merry cells are CANDI-positive. B. frequency of CANDI-633 positive cells in various other tissues as determined by floss cytometry. C. Serial intravital microscopy images of MC 38-TagBFP (red) tumors showing the movement of CANDI-positive dendritic cells (**D**). DC are marked by white asterixis and are followed over time as shown in panel D (1 h traces of labeled cells). The average speed of the cells was 1.5 ± 0.7 μm/min.

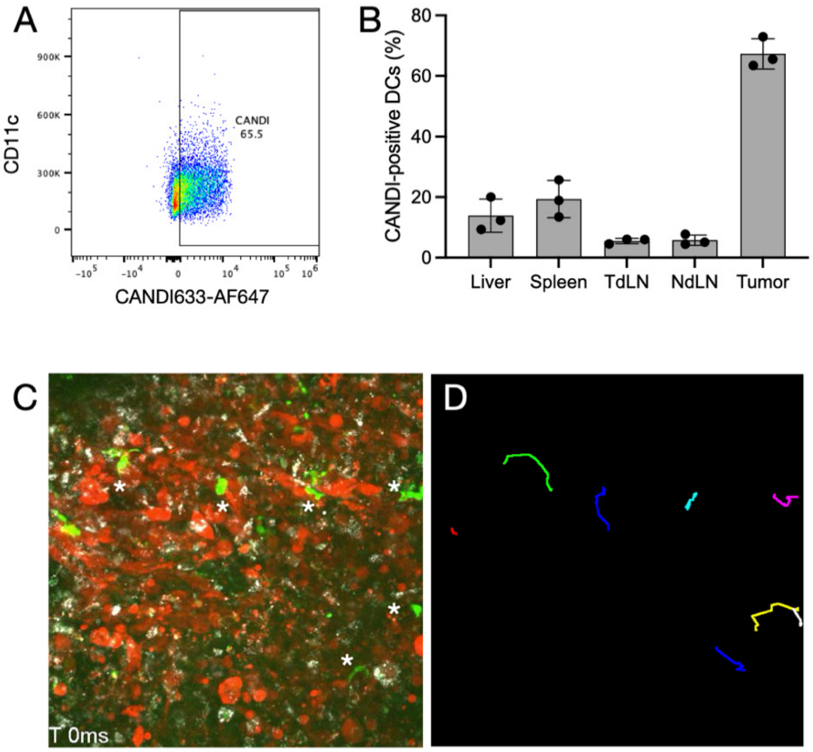

Figure S12: CANDI-633 leads to normalization of tumor vasculature as part of its antitumor effect. Serial intravital images of MC38-TagBFP2 (red) tumor in which the tumor vasculature was imaged over time by IV administration of fluorescent dextran (**A**). Non-fluorescent CANDI633 was administered within 1 h after the baseline images were obtained. Note the remarkable straightening of otherwise chaotic tumor neovasculature 2-3 days after IV administration of CANDI633.

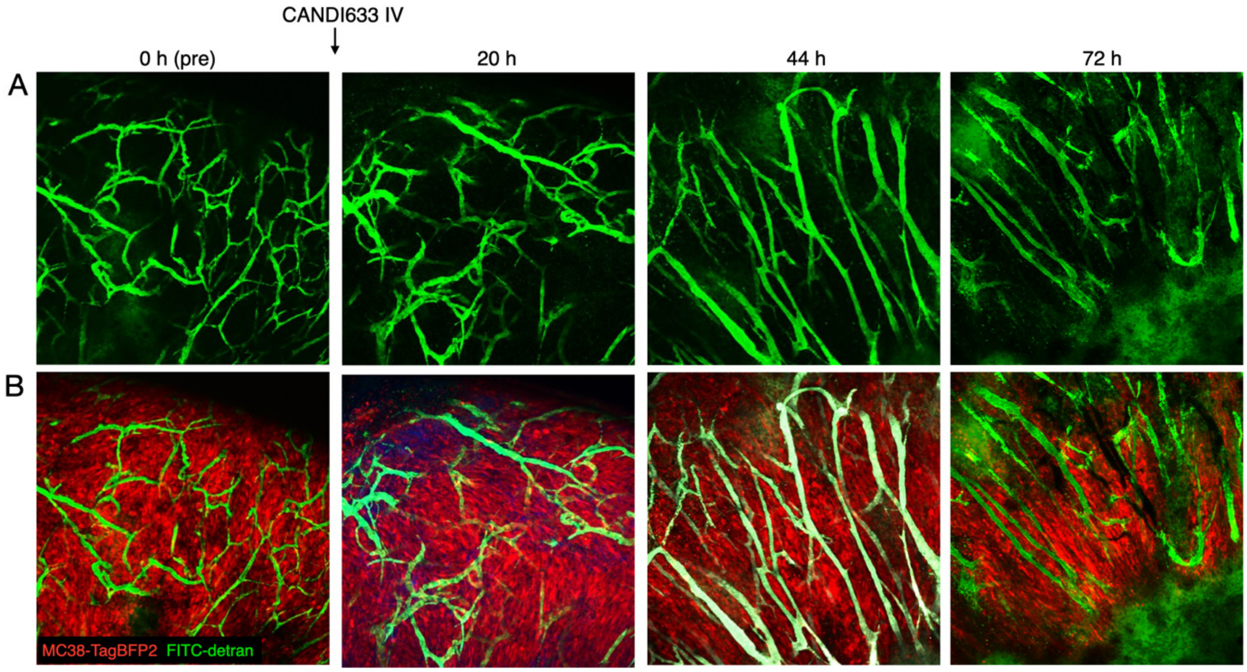

Figure S13: Mechanism. The lead compound containing triple immunostimulants (poly I:C for TLR3 agonism, R848 for TLR 7/8 agonism, and LCL-161 for cIAP inhibition) accumulate in DC and other antigen-presenting cells in the tumor microenvironment and in draining lymph nodes following systemic injection. It is most likely that efficient nanoparticle uptake is mediated by the carbohydrate core, as has been observed for dextrans and dextrins [25,27,32]. Payload delivery in DC up-regulates different cytokines, most importantly IL-12, among others. This leads to DC activation and recruitment of CD8 T cells into the tumor environment.

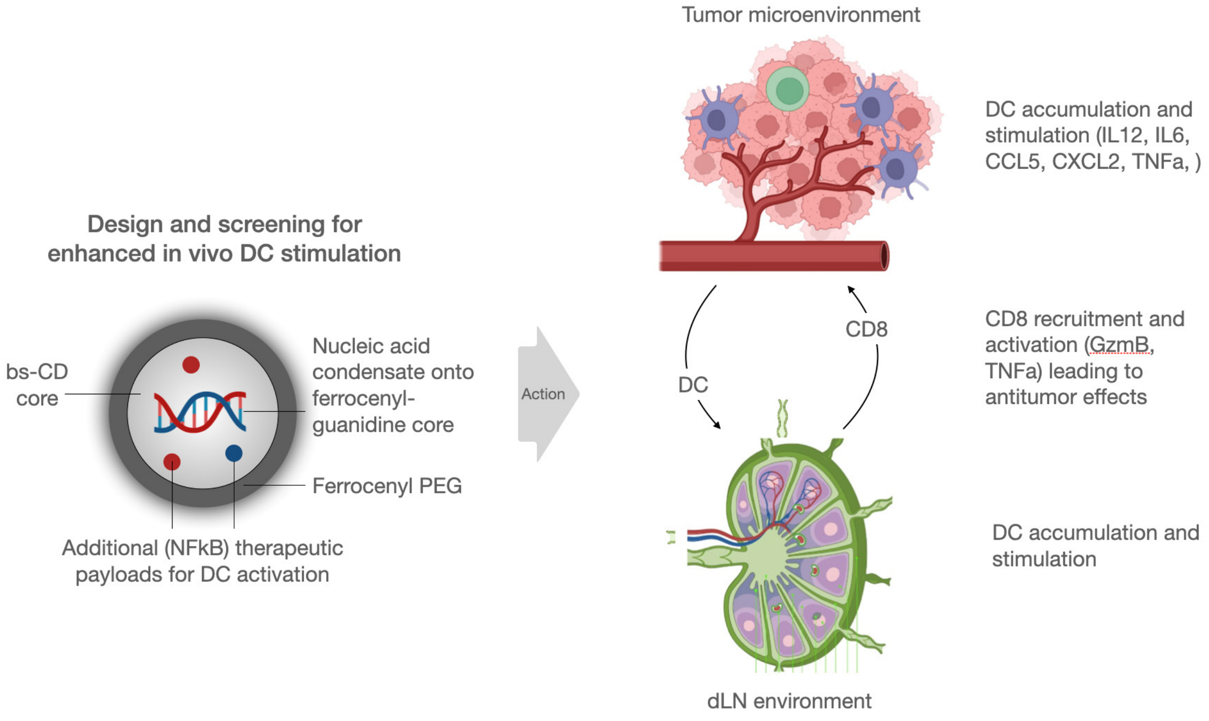

Figure S14: ^{1}HNMR (400 mHz, DMSO*d6*) spectrum of Ferrocene-guanidine (FG).

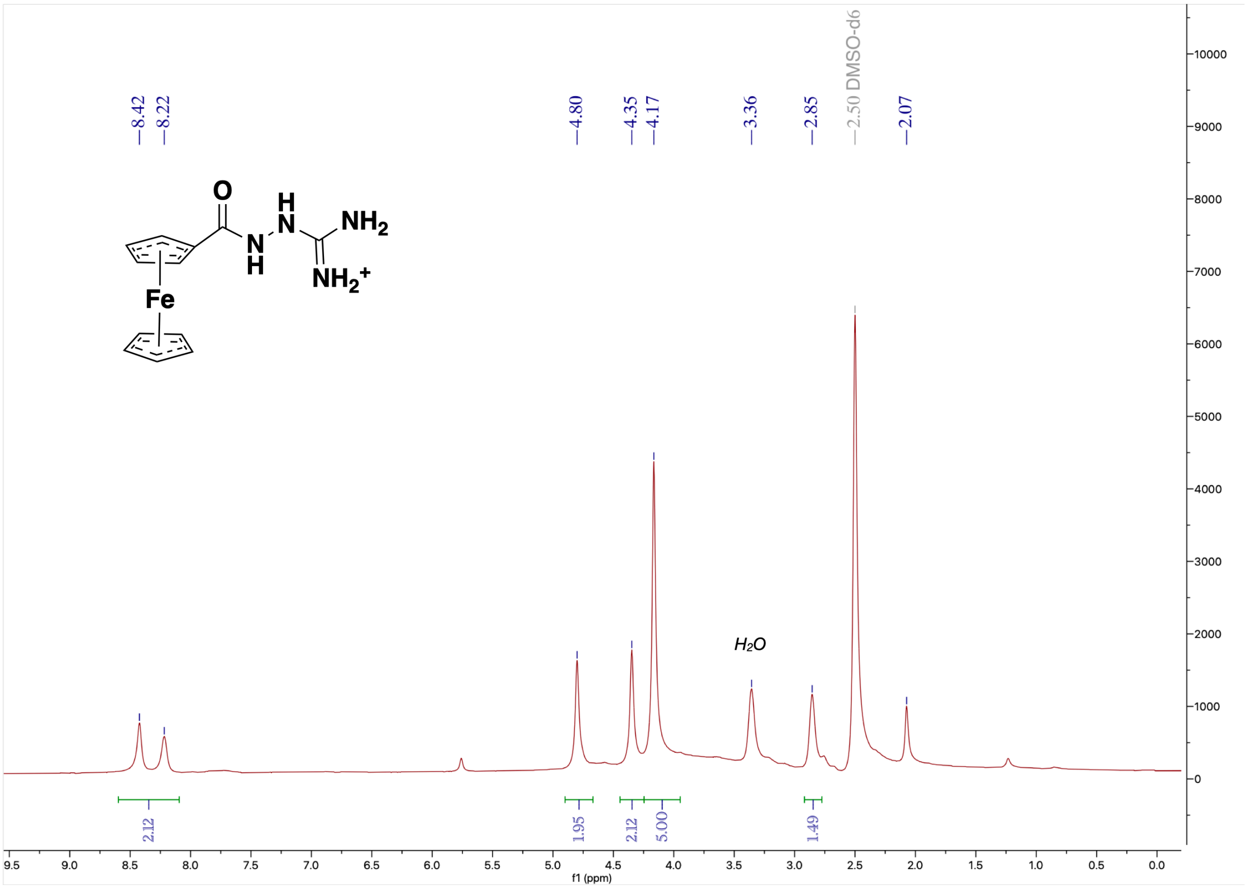

Figure S15: ^{1}HNMR (400 mHz, DMSO$_{d6}$) spectrum of Ferrocene-NHS (FG).

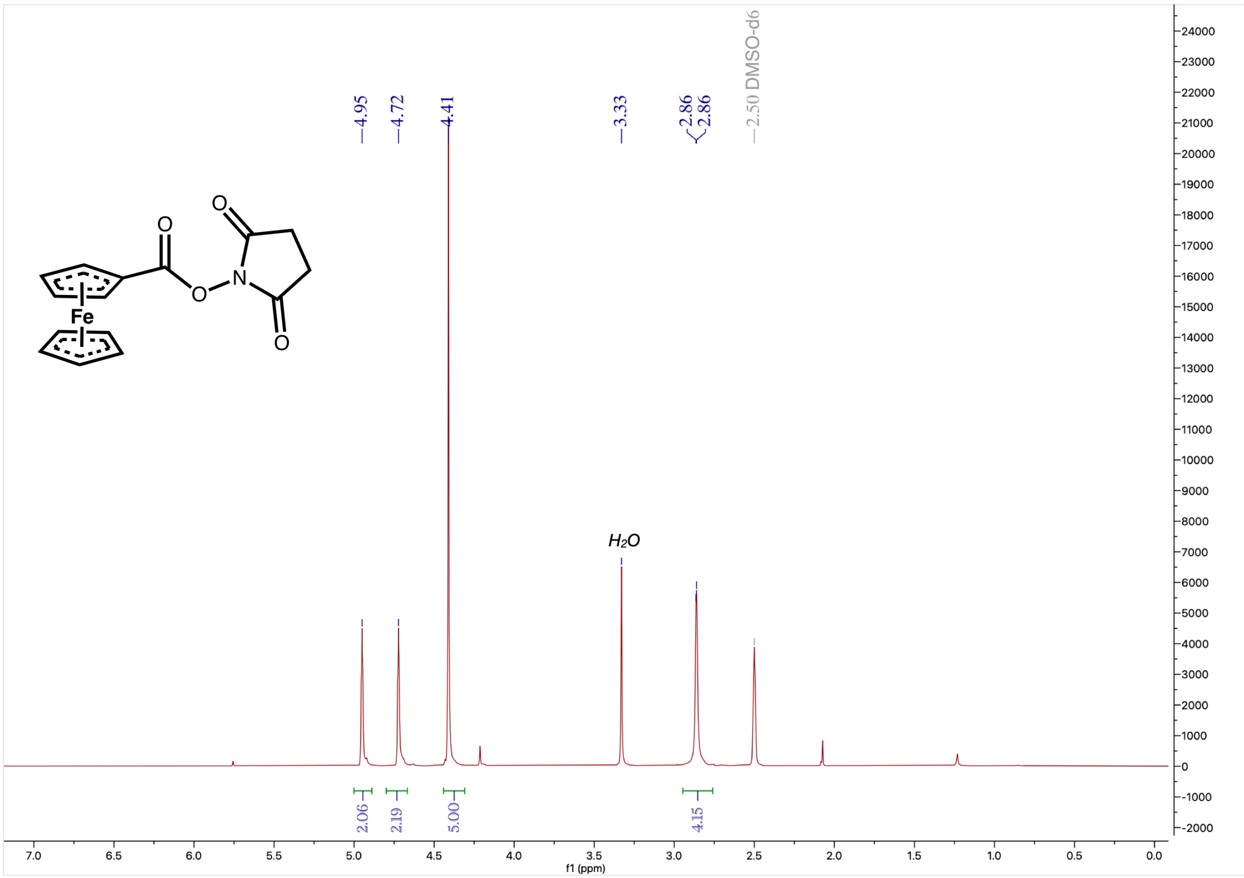

Figure S16: A.^{1}HNMR (400 mHz, D_2O) spectrum of Ferrocene-PEG (FG). **B**. Two-Dimensional 1H-COSY NMR spectrum showing the complex H-coupling in the aliphatic region (0–5 ppm) and the significant signal overlap of the ferrocene moiety with the large PEG functional groups (3.5 ppm). C.^{13}CNMR (100 mHz, D_2O) spectrum of Ferrocene-PEG (FG).

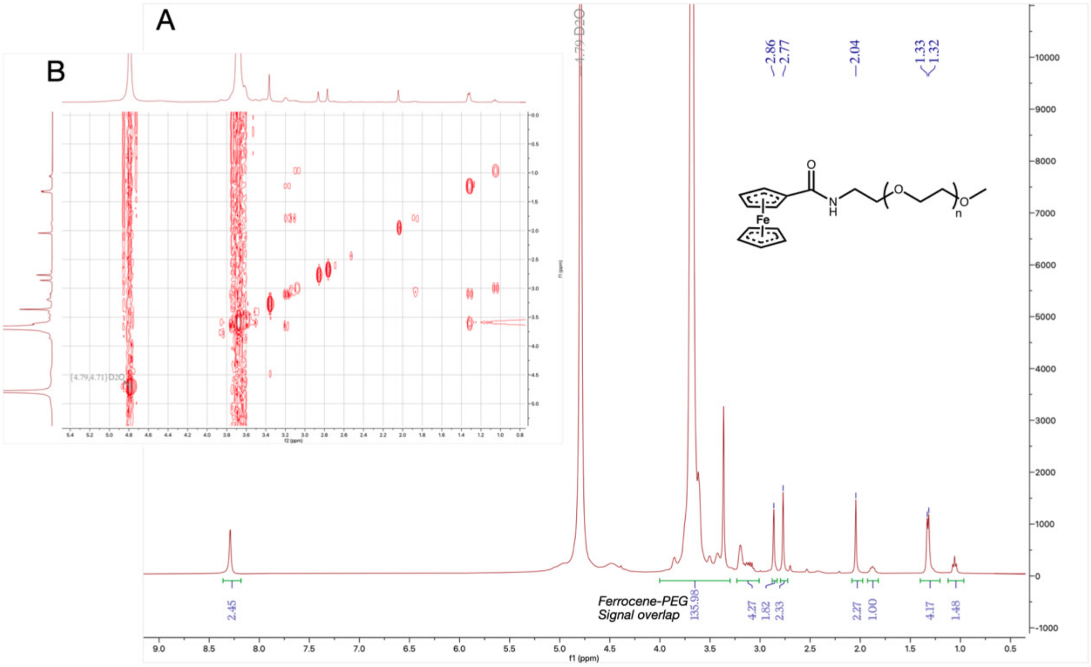

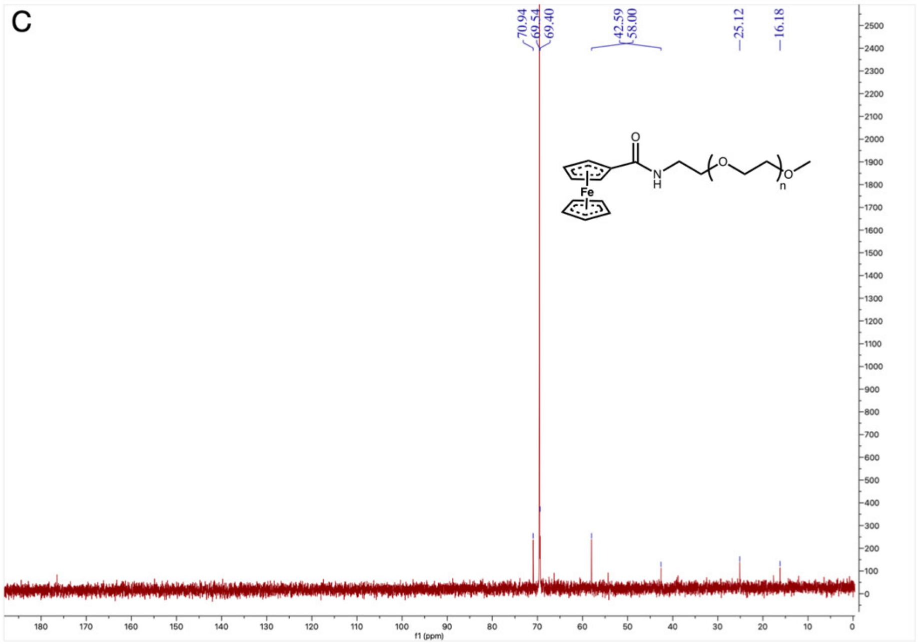

Table. S1: Overview of nanoconstructs synthesized.
Shown are the mean sizes polydispersity indices (PDI) of single batches. NA: nucleic acid. Payload: small molecule payload in the core. FG: ferrocenyl guanidine. FPEG: ferrocenyl PEG. NAPED: *N*[1]-(2-(4-(2-Aminoethyl) piperazin-1-yl)ethyl) ethane-1,2-diamine

Code	Other name	Size, nm	PDI	NA	Payload	Comments
600	Crosslinker: NAPED	18.1 ± 3.5	0.263	None	None	Core only
601	Crosslinker: Betaine	18.8 ± 2.1	0.671	None	None	Core only
602	Crosslinker: Arginine	60.7 ± 4.3	0.564	None	None	Core only
603	Crosslinker: Triethanolamine	32.7 ± 4.3	0.718	None	None	Core only
604	Crosslinker: Diethanolamine	35.4 ± 2.5	0.538	None	None	Core only
605	Crosslinker: Ethanolamine	25.8 ± 1.4	0.877	None	None	Core only
606	Crosslinker: Phosphoserine	228.2 ± 43.2	0.283	None	None	Core only
607	Crosslinker: Serine	38.5 ± 5.3	0.507	None	None	Core only
608	Crosslinker: Lysine	38.9 ± 4.2	0.430	None	None	Core only
609	Crosslinker: Taurine	146.7 ± 13.1	0.367	None	None	Core only
610	NAPED-FG	20.9 ± 2.4	0.232	None	None	Core with FG
611	Betaine-FG	25.4 ± 2.1	0.287	None	None	Core with FG
612	Arginine-FG	75.4 ± 0.6	0.205	None	None	Core with FG
613	Triethanolamine-FG	89.9 ± 3.2	0.216	None	None	Core with FG
614	Diethanolamine-FG	82.6 ± 1.1	0.240	None	None	Core with FG
615	Ethanolamine-FG	194.0 ± 3.2	0.306	None	None	Core with FG
616	Phosphoserine-FG	190.4 ± 32.2	0.630	None	None	Core with FG
617	Serine-FG	91.9 ± 2.5	0.233	None	None	Core with FG
618	Lysine-FG	91.8 ± 2.1	0.187	None	None	Core with FG
619	Taurine-FG	16.7 ± 2.4	0.553	None	None	Core with FG
620	NAPED-FG-FPEG	23.3 ± 2.6	0.283	None	None	Core with FG and FPEG
621	cGAMP	45.7 ± 3.2	0.288	CDN	None	NA loading
622	mRNA	44.7 ± 2.4	0.304	mRNA	None	NA loading
623	Poly I:C_{Low}	99.8 ± 12.4	0.063	Poly I:C	None	NA loading
624	pDNA	101.5 ± 4.5	0.14	DNA	None	NA loading
625	Poly I:C_{High}	151.9 ± 5.3	0.122	Poly I:C	None	NA loading
631	Mono lead	109 ± 8.7	0.183	Poly I:C^{low}	LCL161	Dual SM loading
632	Mono lead	110 ± 6.3	0.188	Poly I:C^{low}	R848	Dual SM loading
633	Final Lead compound (LMW)	114 ± 8.7	0.229	Poly I:C^{low}	R848 + LCL161	Triple SM loading
634	Mono lead	144.6 ± 6.4	0.222	Poly I:C^{High}	LCL161	Dual SM loading
635	Mono lead	146.8 ± 4.3	0.264	Poly I:C^{High}	R848	Dual SM loading
636	Alt Lead compound (HMW)	162 ± 6.7	0.287	Poly I:C^{High}	R848 + LCL161	Triple SM loading
